# Therapeutic Potential of *Centella asiatica* and Its Triterpenes: A Review

**DOI:** 10.3389/fphar.2020.568032

**Published:** 2020-09-04

**Authors:** Boju Sun, Lili Wu, You Wu, Chengfei Zhang, Lingling Qin, Misa Hayashi, Maya Kudo, Ming Gao, Tonghua Liu

**Affiliations:** ^1^Second Clinical Medical College, Beijing University of Chinese Medicine, Beijing, China; ^2^Key Laboratory of Health Cultivation of the Ministry of Education, Beijing University of Chinese Medicine, Beijing, China; ^3^Technology Department, Beijing University of Chinese Medicine, Beijing, China; ^4^School of Pharmaceutical Sciences, Mukogawa Women’s University, Hyogo, Japan

**Keywords:** anti-apoptosis, anti-inflammatory, anti-oxidative stress, *Centella asiatica*, triterpenoids

## Abstract

*Centella asiatica* (also known as *Centella asiatica* (L.) Urb. or Gotu kola) is a traditional Chinese medicine with extensive medicinal value, which is commonly used in Southeast Asian countries. This study aimed to summarize the effects of *C. asiatica* and its main components on neurological diseases, endocrine diseases, skin diseases, cardiovascular diseases, gastrointestinal diseases, immune diseases, and gynecological diseases, as well as potential molecular mechanisms, to study the pathological mechanism of these diseases based on the changes at the molecular level. The results showed that *C. asiatica* and its triterpenoids had extensive beneficial effects on neurological and skin diseases, which were confirmed through clinical studies. They exhibited anti-inflammatory, anti-oxidative stress, anti-apoptotic effects, and improvement in mitochondrial function. However, further clinical studies are urgently required due to the low level of evidence and lack of patients.

## Introduction

**Figure 1:**
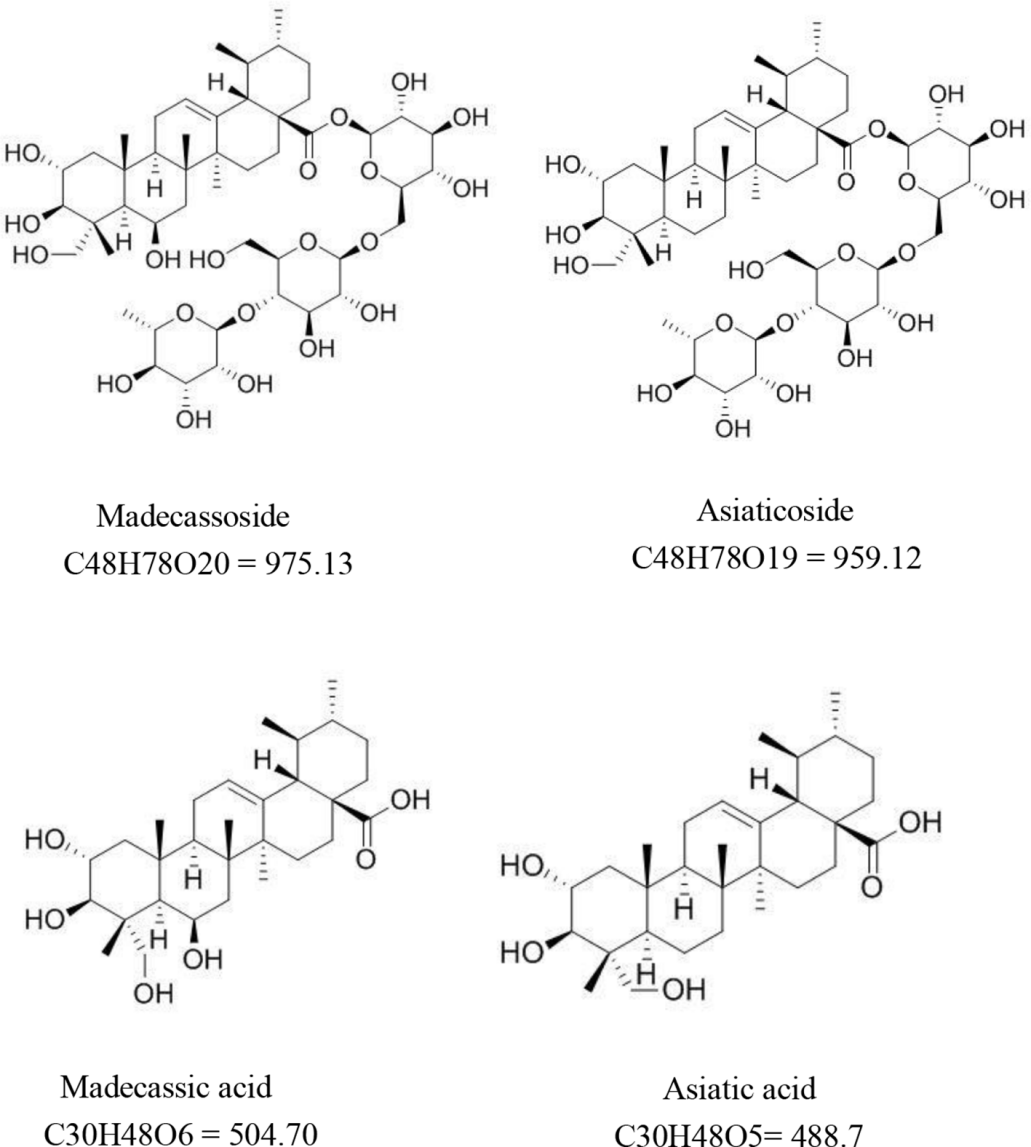
Molecular formulas of several main compounds of *Centella asiatica*.

*Centella asiatica*, also known as *Centella asiatica* (L.) Urb. or Gotu kola, is an herb used in traditional Chinese medicine in China and Southeast Asian countries to treat a variety of diseases. The earliest records of *C. asiatica* in China can be retraced back to “*su wen shi*” and “*zheng lei ben cao*” in Song Dynasty. It is described as follows: “*Centella asiatica*, bitter, cold, nontoxic. Suitable for use in fever and skin conditions.” Another source “*ming jia fang xuan*” lists the herb for use in treating Gilles de la Tourette syndrome in children. A large number of animal and cell experiments have been performed on *C. asiatica* and its active components. *Centella* contains several pentacyclic triterpenoids, including asiaticoside, brahmoside, and madecassic acid, along with other constituents such as centellose, centelloside, and madecassoside (Murray and Pizzorno, 2012; Singh and Rastogi, 1968; Singh and Rastogi, 1969). The main chemical components responsible for its pharmacological activity are triterpenes, mostly asiaticoside, asiatic acid, madecassoside, and madecassic acid (**Figure 1**).

Madecassoside (pubchem CID: 131801373) is a pentacyclic triterpene saponin from *C. asiatica* with multiple pharmaceutical activities. It has a molecular formula of C48H78O20 and a molecular weight of 975.1 g/mol. It is widely distributed in the heart, liver, spleen, lung, brain, stomach, skin, and kidney through oral dosing, reaching maximum levels within 5–15 min after oral administration (Leng et al., 2013; Anukunwithaya et al., 2017). Asiaticoside (pubchem CID: 52912190) has a molecular formula of C48H78O19 and a molecular weight of 959.1 g/mol. It also reaches maximum levels within 5–15 min after oral administration. Asiaticoside is extensively distributed in the brain, stomach, and skin within 1 h after dosing (Anukunwithaya et al., 2017). Madecassic acid (pubchem CID: 73412) has a molecular formula of C30H48O6 and a molecular weight of 504.7 g/mol. It is a pentacyclic triterpenoid in which ursane is substituted by a carboxy group at position 28 and hydroxy groups at positions 2, 3, 6, and 23 (the 2alpha, 3beta, 6beta stereoisomer). A previous study confirmed that madecassic acid was found in the plasma, brain, heart, liver, kidney, colon, and bladder after oral administration (Yin et al., 2012). The molecular formula of asiatic acid (pubchem CID: 119034) is C30H48O5, and the degree of solubility in water is 5.98 × 10^−2^ mg/L at 25°C. Although asiatic acid is mainly absorbed in the jejunum (Yuan et al., 2015), it is also distributed in the plasma, brain, heart, liver, kidney, colon, and bladder (Yin et al., 2012). The results were consistent with the review by Ojha et al. They pointed out that for the physicochemical properties of asiatic acid, it is hardly soluble in water, but stable in saline. Its critical micelle concentration and surface tension are 15 ± 2M and 64.1 mN/m, respectively. Moreover, preclinical and clinical pharmacokinetic data demonstrated that asiatic acid could be distributed in many tissues by binding with albumin. Although the bioavailability of asiatic acid is poor, derivatives of asiatic acid showed multiple therapeutic values. Furthermore, chemical modification of the asiatic acid’s backbone improved its bioavailability and biological activity (**Table 1**) (Lv et al., 2018; Nagoor et al., 2018). Asiatic acid and madecassic acid are biologically active ingredients of glycosides. Although in tissues and plasma their concentration is low, they can be detected in feces within 48 h after oral administration of *C. asiatica* extract. It suggests that triterpenoid glycosides are mainly metabolized in the intestine. (Anukunwithaya et al., 2017). In summary, madecassoside, asiaticoside, madecassic acid, and asiatic acid are widely distributed in the body and madecassoside, asiaticoside may exert their biological activity through converted into aglycone (madecassic acid, and asiatic acid).

**Table 1:** Pharmacokinetic parameters of four triterpenes.

Chemical	Treatment	Dose/Route	Species	C_max_ (µg/L)	T_max_ (h)	AUC _(0–24) (µg×h/L)_	Oral bioavailability (%)	References
Madecassoside	Single	500 mg/p.o	human	5.67 ± 0.62	1	30.12 ± 7.12	Not mentioned	(Songvut et al., 2019)
	Multiple	500 mg/p.o	human	5.23 ± 1.84	1	37.29 ± 16.52	Not mentioned	
	Single	50mg/kg/i.v	rat	NA	NA	1 436 900 ± 562 001	NA	(Anukunwithaya et al., 2017)
	Single	50mg/kg/p.o	rat	1654 ± 884	0.25 ± 0.00	2712 ± 1883	0.19	
Asiaticoside	Single	500 mg/p.o	human	1.50 ± 0.18	1	1.70 ± 0.53	Not mentioned	(Songvut et al., 2019)
	Multiple	500 mg/p.o	human	2.71 ± 1.08	2	11.40 ± 9.67	Not mentioned	
	Single	50mg/kg/i.v	rat	NA	NA	543 530 ± 156 158	NA	(Anukunwithaya et al., 2017)
	Single	50mg/kg/p.o	rat	318 ± 192	0.19 ± 0.10	796 ± 910	0.15	
Madecassic acid	Single	500 mg/p.o	human	52.14 ± 18.67	1.5	357.20 ± 116.65	Not mentioned	(Songvut et al., 2019)
	Multiple	500 mg/p.o	human	80.79 ± 27.76	1.5	681.05 ± 413.17	Not mentioned	
Asiatic acid	Single	500 mg/p.o	human	84.08 ± 33.91	1	724.75 ± 259.98	Not mentioned	(Songvut et al., 2019)
	Multiple	500 mg/p.o	human	116.62 ± 32.26	1	1202.29 ± 293.37	Not mentioned	

p.o, per os; i.v, intravenous administration; C_max_, maximal/peakplasma concentration; T_max_, time to reach peak plasma concentration; AUC, area under the curve; NA, not applicable; Dose, ECa 233 doses.

*C. asiatica* is a traditional Chinese medicine with a wide range of functions. However, to date, the therapeutic effect of this traditional Chinese medicine on multiple diseases has not been systematically reviewed. Therefore, this study retrieved literature on *C. asiatica* and its main components and summarized their impacts on different diseases, so as to understand the broad pharmacological effects of *C. asiatica* comprehensively.

### Methodology

The database PubMed was searched for literature on *C. asiatica* published between January 1, 2015, and October 19, 2019, using the following search terms: (Centella[MeSH Terms]) OR (((((((((((((((((((((Hydrocotyle[Title/Abstract]) OR Hydrocotyles[Title/Abstract]) OR Centella asiatica[Title/Abstract]) OR Centella asiaticas[Title/Abstract]) OR asiatica, Centella[Title/Abstract]) OR Gotu kola[Title/Abstract]) OR Gotu kolas[Title/Abstract]) OR kola, Gotu[Title/Abstract]) OR Mandukaparni[Title/Abstract]) OR Mandukaparnus[Title/Abstract]) OR Hydrocotyle asiatica[Title/Abstract]) OR Hydrocotyle asiaticas[Title/Abstract]) OR asiaticas, Hydrocotyle[Title/Abstract])) OR (Centella asiatica (L.) Urb.[Title/Abstract])) OR (Centella asiatica var. asiatica[Title/Abstract])) OR (Centella asiatica var. crista Makino[Title/Abstract])) OR (acariçoba[Title/Abstract])) OR (artaniyae-hindi[Title/Abstract])) OR (asiatic pennywort[Title/Abstract])) OR (asiatic pennywort herb[Title/Abstract])). The search did not exclude studies based on language or status of the publication.

### Inclusion Criteria

The following types of studies were included: (a) experimental studies; (b) clinical trial; (c) not a case report or a review; and (d) medicine identified as *C. asiatica* or the *C. asiatica* extract.

### Exclusion Criteria

The following types of studies were excluded: (a) full text not available; and (b) treatments combined with other ingredients.

## Results

**Figure 2:**
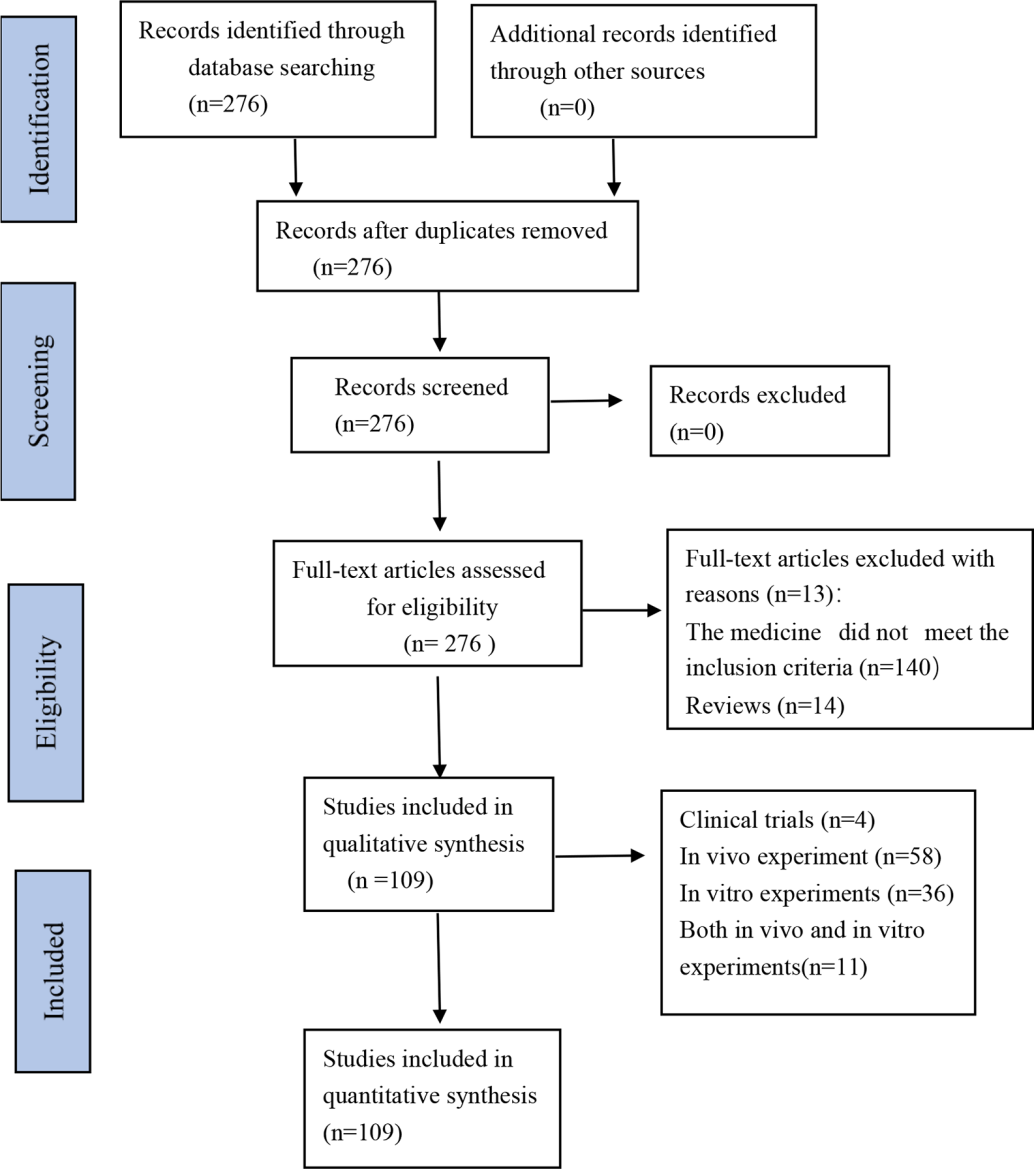
Flow Diagram.

A total of 276 studies were retrieved according to the screening criteria, of which 140 did not meet the inclusion criteria, 14 were excluded because they were reviews, and 13 were excluded for other reasons in full-text study eligibility assessment. Among the 109 studies included, 58 were *in vivo* experiments, 36 *in vitro* experiments, 11 *in vivo* and *in vitro* experiments, and 4 clinical trials (**Figure 2**). Several studies indicated that *C. asiatica* and its triterpenes were effective in many diseases. These diseases were summarized according to different body systems. The pathological mechanisms underlying these diseases were compared, and the mechanisms through which *C. asiatica* and its extracts affected these diseases were summarized. Based on these findings, a systematic review of the effect of *C. asiatica* on systemic diseases and the possible underlying mechanisms was performed.

### Effects on Neurological Diseases

*C. asiatica* enhances the function of the nervous system. It dissolves in methanol, ethanol, and water. Relevant literature on the nervous system demonstrated that *C. asiatica* and its triterpenes could be used to relieve a variety of neurological diseases, but the most researched are improve Alzheimer’s disease (AD) (Song et al., 2018) and Parkinson’s disease (Nataraj et al., 2017b) (**Table 2**). The pathogenesis of AD and Parkinson’s disease involve neuroinflammatory activities (Gelders et al., 2018), oxidative stress (Jiang et al., 2016), mitochondrial dysfunction (Morais and De Strooper, 2010), and dysfunction in brain-derived neurotrophic factor (Mohammadi et al., 2018). Therefore, this study focused on how *C. asiatica* and its triterpenes affected neurological diseases from the aforementioned four aspects.

**Table 2:** Effects on neurological diseases.

Disease model	Type	Solvent composition of extract	Animal/cell	Signaling pathway	Major findings	References
–	*In vitro* and *in vivo*	Methanolic extract of CA	Male Wistar rats	–	MDA ↓, GSH ↑, SOD ↑, AChE↓	(Arora et al., 2018)
–	*In vivo*	Ethanolic extract of CA	Adult male Swiss mice	–	Antinociceptive, anti-inflammatory, and anxiolytic-like	(Oliveira et al., 2017)
Sleep deprivation	*In vivo*	Ethanolic extract of CA	Male LACA mice	–	Gross locomotor activity ↑, anxiety-like behavior ↓, LPO↓, GSH↑, catalase enzyme↑, SOD↑, TNF-α↓, AChE↓	(Chanana and Kumar, 2016)
–	*In vitro* and *in vivo*	Asiatic acid	Male SD rats; hippocampal cell	–	Spatial working memory↑, Ki-67 cells↑, BrdU-positive cells↑	(Welbat et al., 2016)
Cognitive impairments	*In vitro* and *in vivo*	Asiatic acid	Male SD rats; hippocampal cell	–	Ki-67-positive cells ↑, BrdU-positive cells ↑, and cell survival in the dentate gyrus↑	(Chaisawang et al., 2017)
Hemiparkinsonism	*In vivo*	Asiaticoside	Male SD rats	MAPK	SOD↑, LPO↑, CAT↑, GSH↑, dopamine↑, glutamate↑, Syn1↑, Stx1A↑, PI3K↑, PDK1↑, PEBP↓, VMAT2↑, TH ↑, MAPK ↑, BDNF↑, NGF↑	(Gopi and Arambakkam Janardhanam, 2017)
–	*In vivo*	*Centella asiatica* leaf extract	Adult Wistar rats	–	AChE↑, SOD↑, catalase↑, GPx↑,Glutathione reductase ↑, GSH↑, MDA↓	(Chintapanti et al., 2018)
Alzheimer’s disease	*In vivo*	Water extract of CA	Tg2576 mouse	–	Dendritic arborization↑, dendritic spine density↑,	(Gray et al., 2017a)
Alzheimer’s disease	*In vivo*	Water extract of CA	5xFAD female mice	–	Contextual memory ↑, NRF2↑, NQO1↑, GCLC↑, HMOX1↑, PSD95↑, Mt-ND1↑, Mt-CYB↑, Mt-CO1↑, Mt-ATP6↑, mitochondrial respiration↑	(Gray et al., 2018a)
Alzheimer’s disease	*in vitro*	Water extract of CA	Tg2576 mice	–	ROS↓, NRF2↑, GCLC↑, HMOX1↑, NQO1↑, ATP↑, Mt-ND1↑, Mt-ATP6↑, Mt-CO1↑, Mt-CYB↑, oxygen consumption rate↑	(Gray et al., 2017b)
–	*in vivo*	Water extract of CA	Male and female CB6F1 mice	–	Location memory↑, recognition memory↑, learning and executive function↑, synaptic density↑, NRF2↑, porin↑	(Gray et al., 2018a)
–	*in vivo*	Water extract of CA	C57BL/6 mice	–	NRF2↑, NQO1↑, GCLC↑, HMOX1↑, Mt-ND1↑, Mt-ATP6↑, Mt-CO1↑, Mt-CYB↑, PSD95↑	(Gray et al., 2016)
Chronic Stress	*in vivo*	Ethanolic extract of CA	Male SD rats	–	TNF-*α*↓, BDNF↑	(Ar Rochmah et al., 2019)
Alzheimer’s disease	*in vivo*	Ethanolic extract of CA	Male albino Wistar rats	PP2A/GSK-3B	PP2A↑, GSK-3B↓, Bcl-2↑	(Chiroma et al., 2019a)
–	*in vivo*	Ethanolic extract of CA	Male WKY rats	–	AMPAR GluA2↑, AMPAR GluA1↑, NMDAR GluN2B↑	(Wong et al., 2019)
Mild chronic cerebral hypoperfusion	*in vivo*	Standardized extract of CA	Male SD rats	–	dead neurons ↓, learning flexibility and memory ↑	(Thong-Asa et al., 2018)
–	*in vivo*	Asiatic acid	Male SD rats	–	Notch1↑, Ki-67↑, DCX↑	(Sirichoat et al., 2015)
–	*in vivo*	Standardized extract of CA	Male Wistar rats	–	NR2A↑, NR2B↑, BDNF↑,TrkB↑	(Boondam et al., 2019)
–	*in vivo*	Asiatic acid	Male SD rats	–	Notch1↑, SOX2↑, DCX↑, Nrf2↑, nestin↑, p21 positive cells↓, MDA↓	(Welbat et al., 2018)
Alzheimer’s disease	*in vivo*	Asiatic acid	Male albino Wistar rats	NF-κB	APP↓, Aβ1-42↓, β-,γ- secretases↓, GFAP↓, IL-1β↓, IL-2,4,6↓, TNF-α↓, iNOS↓, COX-2↓	(Rather et al., 2018a)
Parkinson’s disease	*in vivo*	Asiatic acid	Male C57BL/6 mice	PI3K/Akt/mTOR	DAT↑, VMAT-2↑, p-JNK↓, p- P38↓, BDNF↑, GDNF ↑, VEGF↑, TrkB↑, p-PI3K↑, p-Akt↑, p-GSK3β↑, p-mTOR↑, p- p70S6K↑	(Nataraj et al., 2017b)
Alzheimer’s disease	*in vivo*	CA standard extract	Male albino Wistar rats	–	Alleviated the cognitive impairments	(Chiroma et al., 2019b)
Sleep restriction, paradoxical sleep deprivation	*In vivo*	Aqueous solutionso of Hb	Swiss nulliparous female mice	–	Learning↑, memory ↑, AChE↓	(Barbosa et al., 2019)
–	*In vivo*	Asiaticoside-D	Worms	–	MAO-A↓, MAO-B↓	(Subaraja and Vanisree, 2019)
Alzheimer’s disease	*In vivo*	Asiatic acid	Male albino Wistar rats	Akt/GSK-3β	GSH↑, SOD↓, catalase↑, GPx↑, Bcl-2↑, Bax↓, cyto-c↑, caspases-3, 6, 8, and 9↓, CDK5↓, Tau↓, p-Akt↑, p-GSK-3β↑	(Ahmad Rather et al., 2019)
–	*In vivo*	Ethanolic extract of CA	Albino male Wistar rats	–	AMPA GluA1 receptor↑	(Binti Mohd Yusuf Yeo et al., 2018)
Alzheimer’s disease	*in vitro*	Asiatic acid	Human neuroblastoma SH-SY 5Y cell	Akt/GSK3β	ROS↓, Bcl-2↑, Bax↓, cytoc(M)↑, p-AKT↑, p-GSK3β↑, Caspases-3↓, Caspases-9↓	(Rather et al., 2018b)
–	*in vitro*	Asiatic acid	BV2 microglia cells	Sirt1/NF-κB	NF-κB p65↓, NO↓, iNOS↓, TNF-α↓, IL-1β↓, IL-6↓	(Qian et al., 2018)
Alzheimer’s disease	*in vitro*	Asiaticoside	Human brain microvascular endothelial cells	TLR4/NF-kB	TNF-α↓, IL-6↓, TLR4↓, TRAF6↓, p- p65↓	(Song et al., 2018)
Alzheimer’s disease	*in vitro*	Water extract of CA	MC65 and the SH-SY5Y neuroblastoma cell	–	Porin↑, ATP↑, intracellular calcium↓, Mt-ND1↑, Mt-CYB↑, Mt-CO1↑, Mt-ATP6↑	(Gray et al., 2015)
Alzheimer’s disease	*in vitro*	Ethanolic extract of CA	Rat PC12 pheochromocytoma cells and human IMR32 neuroblastoma Cells	–	SOD↑, catalase↑, GR↑, GPx↑, GSH/GSSG ↑	(Chen et al., 2016)
–	*in vitro*	Madecassoside	BV2 microglia cells	NF-κB	ROS↓, iNOS↓, COX−2↓, HO-1↓, STAT1↓, NF−κB↓	(Sasmita et al., 2018)
Cerebral ischemia	*in vitro*	Asiaticoside	Ischemia hypoxia cell	–	Lactate dehydrogenase↓, Bcl-2↑, Bax↓, caspase-3 ↓	(Sun et al., 2015)
Parkinson’s disease	*in vitro*	Asiatic acid	Human neuroblastoma (SH-SY5Y) cell lines	–	ROS↓, MMP↑, Bcl-2↑, Bax↓, caspases-3, -6, -8, and -9↓, cyt c↑	(Nataraj et al., 2017a)
–	*in vitro*	Water extract of CA	Human mesenchymal stem cell	–	Proliferation↓, S100β↑, p75 NGFR↑, MOG↑, GFAP↑	(Omar et al., 2019)

CA, Centella asiatica; MDA, malondialdehyde; GSH, glutathione; SOD, superoxide dismutase; AChE, acetylcholinesterase; LPO, lipid peroxidase;TNF-α, tumour necrosis factor-α; CAT, catalase; Syn1, synaptojanin 1; Stx1A, syntaxin 1A; PI3K, phosphoinositide 3-kinase; PDK1, phosphoinositide-dependent kinase-1; PEBP, phosphatidylethanolamine binding protein; VMAT2, vesicular monoamine transporter 2; TH, tyrosine hydroxylase; MAPK, mitogen-activated protein kinase; BDNF, brain-derived neurotrophic factor; NGF, nerve growth factor; GPx, glutathione peroxidase; NRF2, Nuclear factor(erythroid-derived2)-like2;NQO1, NAD(P)H dehydrogenase-quinone oxidoreductase 1; GCLC, glutamate-cysteine ligase, catalytic subunit; HMOX1, heme oxygenase 1; PSD95, post-synaptic density protein 95 ;Mt-ND1, mitochondrially encoded NADH dehydrogenase 1;Mt-CYB, mitochondrially encoded cytochrome B;Mt-CO1, mitochondrially encoded cytochrome c oxidase 1; Mt-ATP6, mitochondrially encoded ATP synthase 6; ROS, reactive oxygen species; PP2A, protein phosphatase 2 ;GSK-3B, glycogen synthase kinase-3 beta; Bcl-2, B-cell lymphoma 2; AMPAR, α-amino-3-hydroxy-5-methyl-4-isoxazolepropionic acid receptor; NMDAR, N-methyl-D-aspartate receptor; DCX, doublecortin; TrkB, tyrosine kinase B; SOX2, sex determining region Y-box 2; Nrf2, nuclear factor erythroid 2-related factor 2; APP, amyloid precursor protein; GFAP, anti-glial fibrillary acidic protein; IL, interleukins; iNOS, nitric oxide synthase; COX-2, cyclooxygenase- 2; DAT, Dopamine transporter; VMAT-2, Vesicular monoamine transporter-2; JNK, c-Jun N-terminal kinase-p53-Bax; GDNF, Glial cell line derived neurotophic factor; VEGF, Vascular endothelial growth factor; GSK3β, Glycogen synthase kinase 3β; MAO, monoamine oxidase; CDK5, cyclin-dependent kinase 5; NO, nitric oxide; MMP, metalloproteinase; TLR4,Toll-like receptor 4; GR, glutathione reductase; GSSG, glutathione disulphide; HO−1, heme oxygenase 1; STAT1, Signal transducer and activator of transcription 1; NGFR, nerve growth factor receptor ; GFAP, glial fibrillary acidic protein; MBP, myelin binding protein.↑, increase significantly;↓,decrease significantly;-,not mentioned.

First and foremost, the inflammatory cytokines produced by neuroinflammation are closely related to the occurrence of neurodegenerative lesions, which are manifested in AD by affecting the expression and metabolism of amyloid precursor protein (Alasmari et al., 2018). The main pathological change of AD is characterized by the accumulation of beta-amyloid (Aβ)-containing neuritic plaques and neurofibrillary tangles (Yuan et al., 2020). Neuroinflammation is also crucial pathogenesis for Parkinson’s disease (Chen Z. et al., 2018). The chronic increase of pro-inflammatory mediators induces neurotoxic Aβ, plaque formation in AD, and induces neurodegeneration in PD. These pro-inflammatory mediators further aggravate neuroinflammation by recruiting immune cells to the brain. Neuroinflammation affect cells proliferation and maturation through pro-inflammatory cytokines, leading to synaptic dysfunction and neuronal death; thus, are responsible for effectuation of AD and PD (Kempuraj et al., 2017; Martinez-Cue and Rueda, 2020).

Second, oxidative stress refers to a state of imbalance between oxidative and antioxidant effects in the body; it is considered to be an important factor leading to aging. Increased production of reactive oxygen species (ROS) can directly affect neuronal synaptic activity and neurotransmission, leading to cognitive dysfunction. Under normal conditions, superoxide dismutase (SOD), glutathione peroxidase (GPX), and catalase can act as free radical scavengers, affecting the level of ROS. The activation of nuclear factor erythroid-2-related factor 2 (Nrf2) prevents oxidative stress (Tönnies and Trushina, 2017). Previous studies found that *C. asiatica* and its triterpenoids could effectively increase SOD and GPX activities, activate nuclear factor erythroid-2-related factor 2, improve the cognitive impairment of animals, and then alleviate the symptoms of related diseases (Gray et al., 2017a; Chintapanti et al., 2018; Welbat et al., 2018).

Third, mitochondria are the main place where cells carry out aerobic respiration. Mitochondrial dysfunction is closely related to the occurrence of AD and Parkinson’s disease. The signaling pathway of cell death can be activated by mitochondrial ROS. Hence, restoring mitochondrial dysfunction can recover neuronal function in AD and Parkinson’s disease (Onyango et al., 2017). The results showed that *C. asiatica* and its triterpenoids could reduce ROS production (Gray et al., 2017a; Nataraj et al., 2017a).

Last but not least, brain-derived neurotrophic factor is closely related to neuron maintenance, neuron survival, and neurotransmitter regulation. The concentration of this factor is reduced in the brain of patients with neurodegenerative diseases (Lima Giacobbo et al., 2019). *C. asiatica* extract, asiatic acid, and asiaticoside could effectively increase the content of brain-derived neurotrophic factor (BDNF) (Gopi and Arambakkam Janardhanam, 2017; Nataraj et al., 2017b; Chintapanti et al., 2018; Boondam et al., 2019).

*C. asiatica* and its triterpenoids affect neurological diseases possibly through the mitogen-activated protein kinase (MAPK) signaling pathway, phosphotidylinositol 3 kinase/protein kinase B/mammalian target of rapamycin (PI3K/Akt/mTOR)signaling pathway, and nuclear factor kappa-light-chain-enhancer of activated B cells (NF-kB) signaling pathway (**Table 2**). The MAPK signaling pathway is activated by a variety of extracellular stimuli, including growth factors, mitogens, hormones, cytokines, and different cellular stress factors (such as oxidative stress). Also, the p38 MAPK signaling pathway can modulate various events regarding AD, such as tau phosphorylation, neurotoxicity, neuroinflammation, and synaptic dysfunction (Lee and Kim, 2017) The PI3K/Akt/mTOR pathway is a major intracellular signaling pathway that regulates the cell cycle. It is directly related to cellular quiescence, proliferation, and longevity. An *in vivo* study found that the inhibition of the PI3K/Akt/mTOR signaling pathway led to a decrease in the expression of c-Jun N-terminal kinase-p53-Bax 3(JNK3), thus protecting dopaminergic neurons and improving Parkinson’s disease (Chen Y. et al., 2018). Moreover, ROS can mediate the PI3K/Akt/mTOR signaling pathway to exert related effects (Chen et al., 2017). NF-κB is a protein complex that controls cytokine production, cell survival, and transcription of DNA. This signaling pathway is implicated in the process of many diseases of the brain (Saggu et al., 2016; Caviedes et al., 2017).

In conclusion, *C. asiatica* and its extracts had a positive effect on diseases of the nervous system. More importantly, *C. asiatica* and its extracts improved neurological diseases by reducing inflammatory factors, balancing oxidative stress, repairing abnormal expression of mitochondrial-related proteins, and improving the content of BDNF. In addition, they reduced related nerve cell apoptosis, increased synaptic density, and improved the survival rate of neural cells (Chaisawang et al., 2017; Gray et al., 2018a; Rather et al., 2018b).

### Effects on Endocrine Diseases

*C. asiatica* extracts are promising in treating endocrine diseases, especially type 2 diabetes and obesity (**Table 3**). As for specific compounds, asiatic acid was effective in obesity (Rameshreddy et al., 2018) and madecassoside might be a potential candidate for treating osteolytic bone diseases (Wang et al., 2019).

**Table 3:** Effects on endocrine diseases.

Disease model	Type	Solvent composition of extract	Animal/cell	Signaling pathway	Major findings	References
Type 2 diabetes mellitus	*In vivo*	Methanol extract of CA	Male SD rats	–	Blood glucose ↓, food and water intake ↓, ALT↓, AST↓, PFK ↑, GS ↑, GP↑, glycogen content ↑	(Oyenihi et al., 2019)
Obese-diabetic	*In vivo*	Ethanol extract of CA	Male SD rats	–	Glucose ↓, cholesterol ↓, LDL ↓, choline ↑, succinate ↑, lactate ↑, glycerol ↓, GPC ↓, leucine ↓, isoleucine ↓	(Maulidiani et al., 2016)
Obesity	*In vivo*	Asiatic acid	Male SD rats	–	Body weight ↓, plasma glucose ↓, insulin resistance ↓, leptin ↓, adiponectin ↓, amylase ↓, pancreatic lipase ↓, SOD ↑, CAT ↑, GPX ↑, GSH ↑, CPT 1 ↑, UCP 2 ↑, ACC 1↓	(Rameshreddy et al., 2018)
Type 2 diabetes mellitus	*In vivo*	Methanol extract of CA	Male SD rats	–	MDA↓, GSH ↑, GST ↑, GPX ↑, IL-1β↓, IL-4 ↓, MCP-1 ↓, TNF-α↓, TG↓	(Oyenihi et al., 2017)
Type 2 diabetes mellitus	*In vivo*	Methanol extract of CA	Male SD rats	–	Blood glucose ↓, GSH ↑, GST ↑, GPX ↑, MDA ↓, TNF-α ↓, IFN-γ ↓, IL-10 ↑	(Masola et al., 2018)
Hyperlipidemic	*In vivo*	Water extract of CA	Male Wistar albino rats	–	Body weight ↓, TC ↓, TG ↓, LDL-C ↓, HDL-C ↑, SOD ↑, GSH ↑	(Kumari et al., 2016)
Osteoporosis	*In vitro + in vivo*	Madecassoside	BMMs, estrogen deficiency‐induced osteoporosis mouse model	NF‐κB, MAPK	NFATc1 ↓,c‐Fos ↓, Acp5 ↓, CTSK ↓,VATPase‐d2 ↓, integrin β3 ↓, calcium oscillations ↓, trabecular spacing ↓, osteoclast surface/bone surface (Oc.S/BS) ↓, osteoclast number/bone surface (N.Oc/BS) ↓, bone volume/total volume (BV/TV)↑, Tb.N ↑	(Wang et al., 2019)
Gestational diabetic	*in vitro*	*Centella asiatica*	HUVECs	–	VCAM-1 ↓, ICAM-1 ↓, monocyte adhesion ↓, p44/42 MAPK ↓, NF-kB p65 ↓	(Di Tomo et al., 2015)

ALT, alanine transaminase; AST, aspartate transaminase; PFK, phosphofructokinase; GS, glycogen synthase; GP, glycogen phosphorylase; LDL, low-density lipoprotein; GPC, glycerophosphorylcholine; CPT-1, palmitoyltransferase-1; UCP 2, uncoupling protein-2; ACC, carboxylase; TG, triglycerides; MCP-1, monocyte chemoattractant protein-1; IFN-γ, interferon-γ; TC, total cholesterol; HDL-C, high density lipoprotein cholesterol; NFATc1, nuclear factor of activated T cells, cytoplasmic 1; CTSK, cathepsinK; VCAM-1, vascular cell adhesion molecule-1; ICAM-1, intercellular adhesion molecule-1; NF-kB p65, Nuclear Factor kappa-light-chain-enhancer of activated B cells.

Type 2 diabetes mellitus (T2DM) is a form of diabetes characterized by high blood glucose, insulin resistance, and a weaker insulin-stimulated response in the presence of high blood glucose level (Zheng et al., 2018). Oxidative stress is mainly caused by lipid peroxidation and has been considered as the main indicator of the pathogenesis and development of T2DM. Oxidative stress causes microvascular and macrovascular complications (Rehman and Akash, 2017). In addition, the inflammation response may cause the occurrence of T2DM by inducing insulin resistance. The inflammation response is exacerbated in the presence of hyperglycemia and can, in turn, worsen hyperglycemia. Hence, targeting the inflammation pathway may be a potential strategy to prevent and control diabetes (Lontchi-Yimagou et al., 2013). In 2015, the World Health Organization defined the body mass index more than 30 kg/m^2^ as obesity and 25–30 kg/m^2^ as overweight (World Health Organisation, 2013). Obesity is a risk factor for many diseases, including cardiovascular disease, musculoskeletal muscle disease, and even cancer (Kolb et al., 2016; Ortega et al., 2016; Collins et al., 2018). Furthermore, obesity causes chronic inflammation of the body and inflammation involving multiple organs (e.g., liver, heart, skeletal muscle, and brain) (Saltiel and Olefsky, 2017). Osteoporosis is a bone metabolism disease, manifesting itself in the form of bone loss and structure degradation. Main targets are the middle-aged and elderly people over the age of 50. The occurrence of osteoporosis can be linked to other endocrine morbidities, like diabetes, obesity, thyroid hormone disease (Dolan et al., 2017; Tanaka et al., 2018; Delitala et al., 2020; Zou et al., 2020).

Based on the aforementioned pathological mechanisms, the potential mechanism of action of *C. asiatica* and its triterpenes on the diseases involving the endocrine system was elaborated from two aspects: reduced oxidative stress and exerted anti-inflammatory effect. First, the *C. asiatica* extract seemed to improve the oxidative stress. Both the diabetic animal model and the obesity animal model demonstrated that the *C. asiatica* extract increased the GSH, CAT, and SOD activities, thereby improving the enzyme antioxidant system (Kumari et al., 2016; Masola et al., 2018; Rameshreddy et al., 2018). Second, the results of animal experiments showed that the *C. asiatica* extract could effectively decrease related inflammation factors (TNF-α, IL-1β, and IL-4). At the same time, it also reduced blood glucose and blood lipid levels (Oyenihi et al., 2017; Masola et al., 2018). Besides, the results showed that the extracts of *C. asiatica* lowered food and water intake and body weight, which suggested that *C. asiatica* extract may affect obesity by influencing the feeding center controlled by central nervous system (Halpern et al., 2008; Blanco et al., 2011). Moreover, the potential of asiatic acid as an anti-obesity agent can be proved from the facts that it suppresses weight gain, and enhance the sensitivity of leptin and insulin. At the molecular level, asiatic acid can increase the level of enzymatic antioxidants (CAT, GPx and SOD), reverse the expression of CPT-1 and UCP-2 that are suppressed by high-fat diet. Therefore, it can be deduced that asiatic acid can repair oxidative stress damage caused by obesity, and can also suppress weight gain by promoting fatty acid oxidation (Rameshreddy et al., 2018). The results of madecassoside intervention in a mouse model of osteoporosis, caused by estrogen deficiency and bone marrow monocytes showed that it can inhibit the expression of related genes by affecting the NF-κB and MAPK signaling pathways (NFATc1, c-Fos, Acp5, CTSK, VATPase-d2), inhibits the generation of osteoclasts, weaken the absorption activity of osteoclasts. It can be inferred that madecassoside can be a potential candidate for the treatment of osteoporosis **(**Wang et al., 2019).

In summary, available evidence showed that the *C. asiatica* extract and asiatic acid could (1) lower blood glucose levels, (2) improve insulin resistance, (3) inhibit weight gain (4) ameliorate inflammation, and (5) improve oxidative stress. Besides, madecassoside could improve osteoporosis by weakening the absorption of osteoclasts and reducing osteoclast formation. These results show that the prospects of *C. asiatica* extract and related components (asiatic acid, madecassoside) for the treatment of endocrine diseases such as diabetes, obesity and osteoporosis are excellent.

### Effects on Skin Diseases

The *C. asiatica* extract and its triterpenoids had certain therapeutic and relieving effects on acne, baldness, vitiligo, atopic dermatitis, and wounds (Sawatdee et al., 2016; Choi et al., 2017; Ling et al., 2017; Ju Ho et al., 2018; Shen et al., 2019) (**Table 4**).

**Table 4:** Effects on skin diseases.

Disease model	Type	Solvent composition of extract	Animal/cell	Signaling pathway	Major findings	References
Acne	*In vitro*	Madecassoside	Human dermal fibroblasts, HaCaT and human monocytic cell line THP-1	TLR2/NF-κB	IL-1β ↓, TLR2 ↓, cyto p65 ↑, AQP3 ↑, LOR ↑, IVL ↑, HA ↑, HAS1 ↑, HAS2 ↑, HAS3 ↑, ROS ↓	(Shen et al., 2019)
Baldness	*In vitro*	Titrated extract of CA	Human hair follicle dermal papilla cells	JAK/STAT	Diameters of spheroids ↑, SOCS1 ↓, SOCS3 ↓, STAT5 ↓, STAT3 ↓, ALP ↑, VCAN ↑, BMP2 ↑, NOG ↑	(Choi et al., 2017)
Incision and burn	*In vitro*	ECa 233	HaCaT cells	ERK/MAPK, FAK/Akt	Skin keratinocyte migration ↑, Rac1 ↑, RhoA ↑, p-FAK ↑, p-Ak t↑, p-ERK1/2 ↑, p-p38 ↑	(Singkhorn et al., 2018)
Vitiligo	*In vitro*	Madecassoside	Human melanocytes	–	MMP ↑, [Ca^2+^]I ↓, Vv ↓, Sv ↓, Nv ↑, LC3-II/LC3-I ↑	(Ling et al., 2017)
–	*In vitro + in vivo*	Extract of CA	L-929 mouse fibroblast cells, male SD rats	–	Collagen synthesis ↑, antibacterial, capillary number ↓, granulation thickness ↑, wounds ↓	(Yao et al., 2017)
Atopic dermatitis	*In vitro + in vivo*	Ethanol extract of CA	RAW 264.7 murine macrophages, HR-1 mice	NF-κB	IgE ↓, WBC ↓, neutrophil ↓, Lymphocytes ↓, B cells ↓, dendritic cell ↓, iNOS ↓, COX-2 ↓, TNF-α↓, IL-1β ↓, IκBα ↓, p65 ↓, p50 ↓, IL-8 ↓, IL-4 ↓, IL-13 ↓	(Ju Ho et al., 2018)
Atopic dermatitis	*In vitro + in vivo*	Titrated extract of CA	RAW 264.7 cellsHR-1 mice	NF-κB	Lymph node weight ↓, iNOS ↓, COX-2 ↓, IgE ↓, TNF-α ↓, IL-6 ↓, IL- 1B ↓, p65 ↓, p50 ↓, p-IκBα ↓	(Park et al., 2017)
Skin wound	*In vivo*	Asiaticoside	Male SD rats	–	Skin flap survival ↑, tissue water content ↓, SOD ↑, MDA ↓, TNF-α ↓, IL-6 ↓, neutrophil density ↓, neovascularization ↑, VEGF ↑, TNF-α ↓, IL-6 ↓, IL-1β ↓, Flap blood flow ↑	(Feng et al., 2019)
Tongue wound	*In vivo*	*Centella asiatica* extract	Male SD rats	–	MPO ↓, MDA ↓, degree of reepithelialization ↑, CD31 ↑	(Camacho-Alonso et al., 2019)
Wound	*In vivo*	Methanol extract of CA	Male Wistar rats	–	Wound healing ↑	(Sawatdee et al., 2016)
Wound	*In vivo*	Asiaticoside-rich hydrogel formulation	Rabbits	–	wound size ↓, epithelization period ↑	(Sh Ahmed et al., 2019)

TLR2, toll-like receptor-2; AQP3, aquaporin-3; LOR, loricrin; IVL, involucrin; HA, hyaluronan; HAS, HA synthases; ALP, alkaline phosphatase; VCAN, versican; BMP2, bone morphogenetic protein 2; NOG, noggin; Rac1, Ras‐related C3 botulinum toxin substrate 1; RhoA, Ras homolog gene family, member A; FAK, focal adhesion kinase; Akt, protein kinase B; ERK, extracellular signal‐regulated kinases; Vv, volume density; Sv, surface density; Nv, numeral density; IgE, immunoglobulin E; WBC, white blood cell; MPO, myeloperoxidase.

Acne is one of the most common skin disorders. Studies have pointed out that the activation of vascular endothelial cells and the involvement of inflammation responses are essential for the early stages of the development of acne lesions (Kircik, 2016). Vitiligo is an acquired depigmenting disorder of the skin, and one of the most common skin diseases. Some studies suggested that oxidative stress may be the initial cause of this disease. The main targets of ROS are mitochondria, causing structural and functional changes (Glassman, 2011). Atopic dermatitis, also known as atopic eczema, is a chronic relapsing inflammatory skin condition (Avena-Woods, 2017). This disease occurs due to skin barrier dysfunction, alterations in cell-mediated immune responses, and Immunoglobulin E(IgE)-mediated hypersensitivity (David Boothe et al., 2017). Skin is the largest organ of the human body playing a vital role in maintaining the body’s physiological homeostasis. The appearance of wounds can lead to an imbalance in physiological homeostasis. The stages of wound healing comprise inflammation, proliferation, epithelialization, angiogenesis, remodeling, and scarring (Sorg et al., 2017).

According to the summary report by 1998 committee for veterinary medicinal products, the transdermal absorption of the active ingredients in *C. asiatica* in rats showed that madecassic acid can quickly penetrate the skin barrier, but the dose measured at the drug application point on the skin after 24 h was only 0.06% concentrated as compared to the original dose. The results of asiatic acid were similar to madecassic acid. The high concentration of asiaticoside administered transdermally did not cause any systemic toxicity, but it could cause excessive keratinization of the skin at the application site. Some reports also suggested allergic dermatitis over using *C. asiatica* externally, but none of them reports the exact dose. *C. asiatica* and its triterpenoids are made into different formulations to explore the treatment options for skin diseases, and it has been established that they have a potential role in wound healing and skin inflammation.

First, for wound healing, the present study found that *C. asiatica* and its triterpenoids had a direct wound-healing function. *C. asiatica* extracted with methanol contains 0.12% asiatic acid, 0.54% madecassic acid, 0.25% asiaticoside and 1.02% madecassoside, and was made into a spray with hydroxypropyl-β-cyclodextrin (HP-β-CD), Eudragit E100, glycerin, PEG 400, etc., and the spray of triterpenes content are close to 100% compared with *C. asiatica* extracted, of course, The wound was healed completely without any skin irritation (Sawatdee et al., 2016). Compared with ordinary gauze, the electrospun gelatin membranes containing *C. asiatica* can promote the wound repair process by affecting the proliferation of fibroblasts and collagen synthesis, and are antibacterial as well (Yao et al., 2017). The asiaticoside-rich hydrogel formulation exhibited 40% fast wound healing without any skin irritation as compared to untreated group. Thick epithelial layer and keratin formation can be found, while granulation tissue, fibroblasts and collagen were formed moderately (Sh Ahmed et al., 2019). Cells studies have found that *C. asiatica* standard extract (ECa 233) can affect the formation of filopodia and promote wound healing by activating the FAK, Akt and MAPK signaling pathways (Singkhorn et al., 2018). In above studies, though the vehicles were different, but animal and cell experiments have found that *C. asiatica* and its triterpenoids improved the degree of re-epithelialization, increased the collagen synthesis, reduced the inflammation around wounds and cause no obvious skin irritation.

Second, for the treatment of atopic dermatitis, *C. asiatica* significantly reduced the inflammation response (TNF-α ↓, IL-1β ↓, IL-8 ↓, IL-4 ↓, and IL-13 ↓), and also the local immune response (IgE ↓). Whether titrated extract of *C. asiatica* (TECA) or ethanol extract of *C. asiatica*, both seems to inhibit hyperkeratosis, mast cell and inflammatory cell infiltration. Both of them can inhibit the expression of iNOS and COX-2 and NF -κB activity, it confirms that *C. asiatica* extract may be a promising therapeutic TCM for the treatment of atopic dermatitis (Park et al., 2017; Ju Ho et al., 2018). The effect of madecassoside in the treatment of dermatitis is reflected in reducing the pro-inflammatory cytokines (IL-1β, TLR2), moreover, it can promote the secretion of AQP3, LOR, IVL in HaCaT keratinocytes and the secretion of HA in human skin fibroblasts, thus can significantly enhance skin hydration (Shen et al., 2019).

Third, madecassoside, a specific component of *C. asiatica*, had a certain improvement effect on vitiligo, and the possible mechanism of action was to reduce the oxidative stress response and weaken the damage to mitochondria by oxidative stress [matrix metalloproteinase (MMP) ↑ and [Ca^2+^]i ↓]. In addition, it was found that the LC3-II/LC3-I ratio of melanocytes treated with madecassoside increased significantly, suggesting that it enhances the autophagy activation of the cells, thereby protecting skin cells from physiological and pathological aging damage. (Ling et al., 2017). Lastly, *C. asiatica* also demonstrated a positive activation effect on dermal papilla, improved the viability of dermal papilla cells and increased the expression of characteristic genes related to hair growth in the cells, thus providing good application prospects for baldness (Choi et al., 2017).

Although *C. asiatica* and its triterpenoids have low transdermal absorption rate, current animal experiments and cell experiments have found that they can effectively promote wound healing, reduce skin inflammatory diseases, and seem to have a certain effect on vitiligo and baldness. The mechanism of action of *C. asiatica* and its ingredients in the treatment of skin diseases is mainly anti-inflammation, anti-oxidation, and weakening of the damage to mitochondria by oxidative stress, which was consistent with the pathogenesis of these diseases.

### Effects on Cardiovascular Diseases

*C. asiatica* has a positive effect on cardiovascular diseases. The main components that affect the cardiovascular system are asiaticoside and asiatic acid. Hypertension and atherosclerosis are the mostly studied diseases in involved articles (**Table 5**).

**Table 5:** Effects on cardiovascular diseases.

Disease model	Type	Solvent composition of extract	Animal/cell	Signal pathway	Major findings	References
Pulmonary hypertension	*In vivo+in vitro*	Asiaticoside	Male SD rats, rats’ PASMCs	TGF-β1/Smad	Mean pulmonary artery pressure ↓, right ventricular hypertrophy↓, vessel thickness ↓, media wall thickness ↓, TGF-β1 ↓, TGF-βRII ↓, p-Smad2/3 ↓	(Wang et al., 2015)
Renovascular hypertension	*In vivo*	Asiatic acid	Male SD rats	Ang II/AT _1_R/NADPH oxidase/NF-κB	SP↓, DP↓, MAP↓, HBF↑, HVR↑, Ang II↓, AT1R↓, AT2R↑, O_2_^−^↓, MDA↓, TNF-α↓	(Maneesai et al., 2017)
Transverse aortic constriction	*In vivo*	Asiatic acid	Male C57BL/6 mice	TGF-β1/Smad2/3	Ventricular wall thickness ↓, left ventricular posterior wall diastolic Dimensions ↓, LVEDD ↓, FS ↑, cardiac output ↑, Bax/Bcl-2↓,caspase-9 ↓, caspase-3 ↓, cytochrome c ↓, TGF-β1 ↓, α-SMA ↓, collagen I ↓, TNF-a ↓, IL-6 ↓, NF-kB ↓, JNK ↓, p-mad2/3/Smad2/3 ↓, Smad7 ↑	(Si et al., 2015)
Myocardial ischemia/reperfusion (MI/R) injury	*In vivo*	Asiatic acid	Male SD rats	Akt/GSK-3β	LVEDV ↓, LVESV ↓, HR ↑, LV dp/dtmax ↑, LV dp/dtmin ↑, myocardial infarction size ↓, LDH ↓, CK ↓, p-Akt ↑, p-GSK-3β ↑, plasma glucose ↓, plasma lactate ↓	(Dai et al., 2018)
–	*In vitro*	Asiaticoside	Human umbilical vein endothelial cells (HUVECs/7th passage)	–	Endothelial permeability ↓, ATP ↓, NO ↓, H_2_O_2_ ↓, IL-18 ↓, sICAM-1 ↓, sVCAM-1 ↓, E-selectin ↓	(Jing et al., 2018)
Pulmonary hypertension	*In vivo + In vitro*	Asiaticoside	Male SD rats; HPAECs	PI3K/Akt/eNOS	Mean pulmonary artery pressure ↓, medial wall thickness ↓, Right ventricular hypertrophy ↓, pulmonary arteriole wall thickening ↓, inflammatory cell infiltration ↓, NO ↑, cGMP ↑, p−Akt ↑, p−eNOS ↑, caspase−3 ↓	(Wang X. et al., 2018)
Atherosclerosis	*In vitro*	Asiatic acid	Human aortic endothelial cells (HAECs).	–	F-actin rearrangement ↓, stabilize the F-actin filaments, MLC dephosphorylation ↓, stabilize peripheral diphospho-MLC, VE-cadherin ↑, inhibit redistribution of occludin and ZO-1	(Fong et al., 2019)
Atherogenic	*In vitro*	Asiaticoside	Human aortic endothelial cells		Vascular permeability ↓	(Fong et al., 2015)
Myocardial ischemic/reperfusion injury	*In vitro*	Asiatic Acid	Rat H9c2 Cardiomyocytes	Akt/GSK-3β/HIF-1α	Apoptotic cells ↓, caspase-9 ↓, caspase-3 ↓, Bax/Bcl-2 ↓, MMP ↑, Ca^2+^ ↓, ROS ↓, p-AKT ↑, p-GSK-3β↑, HIF-1α↑	(Huang et al., 2016)
Atherogenesis	*In vitro*	Asiatic acid	Human aortic endothelial cells(HAECs).	NF-κB	sE-selectin ↓, sICAM-1 ↓, p-IκBα ↓, VCAM-1 ↓	(Fong et al., 2016)

TGF-β, transforming growth factor beta; SP, systolic blood pressure; DP, diastolic blood pressure; MAP, mean arterial blood pressure; HBF, hindlimb blood flow; HVR, hindlimb vascular resistance; Ang II, angiotensin II; FS, fractional shortening; LVEDD, left ventricular end-diastolic diameter; a-SMA, a-smooth muscle actin; LVESV, left ventricular end systolic volume; HR, heart rate; LDH, lactate dehydrogenase; CK, creatine kinase; eNOS, endothelial nitric oxide synthase; cGMP, cyclic guanosinc monophosphate; MLC, myosin light chain; ZO-1, zona occludens -1; HIF-1a, hypoxia-inducible factor 1a.

Hypoxic pulmonary hypertension can cause pulmonary arterial changes, including pulmonary arterial stiffness and narrowing. Ventricular changes caused by right ventricular hypertrophy and right ventricular fibrosis affect ventricular function (Wang and Chesler, 2013). Renovascular hypertension is one of the common causes of secondary hypertension. About 90% of cases are due to atherosclerotic renal artery stenosis and often accompanied by severe occlusive diseases of other blood vessels, accounting for poor prognosis (Matuszkiewicz-Rowińska and Wieliczko, 2015). The Ang II/AT1R signaling pathway can regulate a series of intracellular pathways to improve cardiac insufficiency and myocardial remodeling, which is closely associated with the occurrence and development of renal hypertension (Liu et al., 2017). The transverse aortic constriction contributes to the occurrence of cardiac hypertrophy and failure. The common pathological changes are systolic dysfunction and cardiac fibrosis of the heart. Pressure overload triggers the expression of inflammation genes. Inhibiting early inflammation reactions can reduce cardiac remodeling and improve heart function (Suetomi et al., 2018; Richards et al., 2019). Fibrosis is a pivotal player in the development and progression of heart failure, which is controlled by the TGF-β/Smads pathway. Smad2 and Smad3 are the two main downstream regulators of TGF-β1-mediated tissue fibrosis, and Smad7 is a negative feedback regulator (Hu et al., 2018; de Boer et al., 2019). Cardiovascular diseases can cause a variety of pathological changes and affect the development of related pathology by affecting Ang II/AT1R and TGF-β/Smads signaling pathways. Wang X. B. et al. (2015) found that asiaticoside reduced mean pulmonary artery pressure and right ventricular hypertrophy by inhibiting the overexpressed TGF-β1/Smad2/3 signaling pathway in the hypoxia-induced pulmonary hypertension rat model. In 2017, Wang et al. further confirmed that asiaticoside effectively reduced the apoptotic factor (caspase-3), increasing the production of NO by activating the Akt/eNOS pathway. They confirmed that asiaticoside protected pulmonary hypertension by affecting endothelial cell function effect (Wang X. et al., 2018). And asiatic acid has anti-hypertensive and anti-inflammatory effects. In animal models of renovascular hypertension, it can play the role of angiotensin-converting enzyme (ACE)by inhibiting the Ang II–AT1R–Nicotinamide adenine dinucleotide phosphate (NADPH) signaling pathway. Moreover, it can reduce the inflammatory response (TNF-α ↓, phospho-NF-κB ↓, IL-6 ↓) (Si et al., 2015; Maneesai et al., 2017). A clinical prospective, placebo-controlled, randomized, dose range trial found that after 4 weeks of treatment with *C. asiatica* total triterpenes (TTFCA), the capillary filtration rate, ankle circumference and ankle edema of patients with venous hypertension were improved, and the dose range showed that 180 mg/day was most effective in symptoms improvement (De Sanctis et al., 2001).

Atherosclerosis is a disease in which the inside of arteries narrows due to the buildup of plaque, leading to some serious problems such as heart attack, stroke, or even death. Maintaining arterial integrity and retaining endothelial barrier function and normal contraction of smooth muscle can limit the development of atherosclerotic disease (Döring et al., 2017). Asiaticoside has been found to reduce endothelial permeability; it can effectively protect the occurrence of atherosclerosis by lowering the levels of intercellular adhesion molecule-1, vascular cell adhesion molecule-1, and E-selectin. Moreover, it can also reduce the levels of related inflammation factor (IL-18) and has anti-inflammation effects (Fong et al., 2015; Jing et al., 2018). A cell experiment found that asiatic acid reduced atherosclerosis by inhibiting the redistribution of occludin and zona occludens -1(ZO-1). Furthermore, it decreased F-actin rearrangement and myosin light chain (MLC)dephosphorylation (Fong et al., 2019). Clinical studies have shown that after 4 years of intervention in patients with Pycnogenol^®^ 100 mg/day plus *C. asiatica* (100 mg/day), the combined treatment group has reduced plaque progression, reduced oxidative stress, and mild transient brain deficiency as compared to the control group. The incidence of angina events in combined treatment group was less than 3%, while the control group it was 6.25%. Therefore, it can be established that *C. asiatica* may have a role in preventing preclinical atherosclerosis (Belcaro et al., 2015; Belcaro et al., 2017). For improving the inflammation response, reducing oxidative stress and retaining the endothelial barrier function have beneficial effects on the occurrence and development of atherosclerosis.

In myocardial ischemic disease, apoptosis is the main cause of cardiomyocyte death (Li et al., 2020), and asiatic acid could reduce the levels of apoptotic factors (Bax/Bcl-2, caspase-9, caspase-3) and improve cardiomyocyte apoptosis (Si et al., 2015; Huang et al., 2016). It can also improve the fibrotic changes caused by myocardial dysfunction by affecting the TGF-b1-Smad2/3 signaling pathway. In addition, Rosdah et al. (2019) pointed out that after administering *C. asiatica* extract to rats at doses 200mg/kg and 400mg/kg for 21 days, the content of acetylcholine (ACh) in heart was modulated significantly, which might contribute to its cardioprotective effect. A summary of the related literature (**Table 5**) showed that asiatic acid and asiaticoside had beneficial effects on cardiovascular diseases. Basic experiments confirmed that these two triterpenoids effectively improved hypertension, atherosclerosis, and myocardial ischemia.

### Effects on Digestive Diseases

*C. asiatica* and its triterpenoids also have therapeutic effects on digestive disorders, which is mainly reflected by improved liver fibrosis, colitis, and gastric mucosal damage; and even reduced *Helicobacter pylori* gastric colonization (**Table 6**).

**Table 6:** Effects on digestive diseases.

Disease model	Type	Solvent composition of extract	Animal/cell	Signaling pathway	Major findings	References
Liver fibrosis	*In vivo*	Asiatic acid	Male SD rats	PI3K/Akt/mTOR	Bcl-2 ↑, Bax ↓, caspase-3 ↓, α-SMA ↓, p-AKT ↓, p-mTOR ↓, p70S6K ↓, MDA ↓, SOD ↑, GSH-Px ↑, TGF-β1 ↓, Cox-2 ↓, TNF-α ↓, IL-6 ↓, IL-1β ↓	(Wei et al., 2018)
Drug-induced liver toxicity	*In vivo*	ECa233	Adult male Wistar rats	–	TBARS ↓, SOD ↑, CAT ↑	(Intararuchikul et al., 2019)
Acute liver failure	*In vivo*	Madecassoside	Male KM mice	p38/NF-κB, Nrf2/HO-1	TNF-α ↓, IL-1β ↓, IL-6 ↓, SOD ↑, CAT ↑, GPx ↑, COX-2 ↓, NF-κB p65 ↓, p38 MAPK ↓, iNOS ↓, Nrf2 ↑, HO-1 ↑	(Wang W. et al., 2018)
Liver injury	*In vivo*	Ethanol extract of CA	Male SD rats	–	MDA ↓, SOD ↑, GPx ↑, CAT ↑, IL−1β ↓, IL−2 ↓, IL−6 ↓, IL−10 ↓, IL−12 ↓, TNF−α ↓, IFN−γ ↓	(Choi et al., 2016)
Acute pancreatitis	*In vivo + in vitro*	Asiatic acid	Male BALB/c mice ; pancreatic acinar cells	–	Serum amylase ↓, serum lipase ↓, NF-κB p65 ↓, IκB-β ↑, IL-1β ↓, IL-6 ↓, TNF-α ↓, MPO ↓	(Xiao et al., 2017)
Colon carcinogenesis	*In vivo*	Asiatic acid	Male albino Wistar rats	–	TBARS ↓, CD ↓, LOOH ↓, SOD ↑, CAT ↑, cytochromeP450 ↓, cytochrome P4502E1 ↓, GST ↑, β-glucuronidase ↓, mucinase ↓, LPO ↓, GSH ↑, GPx ↑, GR ↑, VitC ↓, VitE ↓	(Siddique et al., 2017)
Colitis	*In vivo + in vitro*	Madecassoside, asiaticoside, madecassic acid, asiatic acid	Female C57BL/6 mice, EL-4 cells	PPARγ/AMPK/ACC1	Th17 cells ↓, IL-17A ↓, IL-17F↓, IL-21↓, IL-22 ↓, Treg cells↑, Foxp3↑, IL-10↑, ACC1↓, CD36 ↑, LPL ↑	(Xu et al., 2017)
Gastric mucosal injury	*In vivo*	Ethanol extract of CA	Male SD rats	–	iNOS ↓, TNF-α ↓, MDA ↓, COX-2 ↓	(Zheng et al., 2016a)
*Helicobacter pylori* infection	*In vivo*	Ethanol extract of CA	Specific pathogen-free male C57BL/6 mice	–	*H. pylori* SS1 gastric colonization↓	(Zheng et al., 2016b)
Hepatocellular carcinoma	*In vitro*	Asiatic acid	HepG2	–	MMP↓, Ca^2+^↑, ATP↓, cytochrome c↑, mitochondrial swelling↓	(Lu et al., 2016)
Colon cancer	*In vitro*	Asiatic acid	SW480 and HCT116	PI3K/Akt/mTOR/p70S6K	E–cadherin↑, vimentin ↓, N−cadherin↓, p−PI3K↓, p−Akt↓, p−mTOR↓, p−p70S6K↓	(Hao et al., 2018)
Gastric ulcers	*In vitro*	Pentacyclic triterpene–rich *Centella* extract (PRE)	Human gastric epithelialcell lines	–	Cell viability↑, cell migration↑, wound closure↑	(Wannasarit et al., 2019)

TBARS, thiobarbituric acid reactive substances; CD, conjugated dienes; LOOH, lipid hydroperoxides; LPO, lipid peroxidation; GR, glutathione reductase; LPL, lipoprotein lipase.

Chronic and recurrent liver injuries are often accompanied by inflammatory reactions and often develop into liver fibrosis. Therefore, treating chronic and uncontrolled inflammation is a strategy to prevent liver injury and fibrosis (Campana and Iredale, 2017; Nguyen-Lefebvre et al., 2018). The pathological mechanism of gastric mucosal injury is complex, and nonsteroidal anti-inflammatory drugs are relatively common causes (Soll et al., 1991). Prostaglandin biosynthesis is one of the basic components that maintain the integrity of gastric mucosa, and cyclooxygenase is essential in the process of prostaglandin synthesis. The malondialdehyde (MDA) level can reflect the ROS level (Kwiecien et al., 2012). Therefore, an oxidative stress response is also crucial in the process of gastric mucosal injury. The present study found that the *C. asiatica* extract effectively ameliorated the drug-induced liver toxicity, improved gastric mucosal injury, and reduced *H. pylori* infection. The mechanisms involved were as follows: the reduction of related inflammation factors (IL−1β ↓, IL−2 ↓, IL−6 ↓, IL−10 ↓, IL−12 ↓, and TNF−α ↓) and the increase in the level of antioxidant stress factors (SOD ↑, CAT ↑, and GPx ↑). Furthermore, evidence showed that *C. asiatica* could also reduce MDA and COX-2 levels, thereby ameliorating gastric mucosal damage (Choi et al., 2016; Zheng et al., 2016a; Zheng et al., 2016b; Intararuchikul et al., 2019; Wannasarit et al., 2019). The pharmacological effect of asiatic acid is mainly reflected in the improvement in liver fibrosis and acute pancreatitis. It also has a certain therapeutic effect on gastrointestinal tumors. Three mechanisms are reported in the studies: asiatic acid can reduce the level of pro-apoptotic factors (B-cell lymphoma 2(Bcl-2) ↑, Bcl-2-associated X protein(Bax) ↓, caspase-3 ↓), and related inflammation factors (TGF-β1 ↓, TNF-α ↓, IL-6 ↓, IL-1β ↓), and increase the level of anti-oxidative stress factors (SOD ↑, GSH-Px ↑, CAT ↑, GST ↑, GSH ↑) (Xiao et al., 2017; Siddique et al., 2017; Wei et al., 2018.). The study on madecassoside found that it could ameliorate drug-induced acute liver failure by reducing inflammation (TNF-α ↓, IL-1β ↓, IL-6 ↓, iNOS ↓, COX-2 ↓) and oxidative stress (SOD ↑, CAT ↑, GPx ↑, Nrf2 ↑, HO-1 ↑) (Wang W. et al., 2018). Oral administration of the four components of *C. asiatica* could attenuate colitis in mice, but it’s mainly madecassoside acid, the active form of madecassoside, when topically administered in the colon, weakened colitis by regulating Th17/Treg balance *via* affecting the PPARγ/AMPK/ACC1 pathway (Xu et al., 2017).

Colon cancer and primary liver cancer are common types of cancers in the digestive system (Grandhi et al., 2016; Chen et al., 2020). The expression of E-cadherin and vimentin is considered of high reference value in the prognosis of colon cancer. The mitochondrial morphology and the cytosolic calcium level [Ca^2+^] are indicators of the pathological development of hepatocellular carcinoma (Zhang et al., 2011; Huang et al., 2017). Asiatic acid can also affect the expression of epithelial-mesenchymal transition marker proteins in colon cancer cells (E cadherin↑, vimentin ↓, N-cadherin↓), achieved this anti-cancer potential by regulating PI3K/Akt/mTOR/p70S6K signaling pathway. In addition, asiatic acid can induce the dissipation of mitochondrial membrane potential (MMP), ATP depletion, release of cytochrome c from mitochondria into the cytosol of HepG2 cells, which may induce the death of liver cancer cells by directly affecting mitochondrial function, it may be a potential therapeutic drug for liver cancer and colon cancer (Lu et al., 2016; Hao et al., 2018).

Based on the aforementioned evidence, it was concluded that *C. asiatica* maybe can improve liver, colon and stomach related digestive disorders by reducing inflammation, ameliorating oxidative stress, and improving mitochondrial function.

### Effects on Respiratory Diseases

The effects of *C. asiatica* on respiratory diseases is mainly reflected in its ability to improve pulmonary fibrosis, ameliorate chronic obstructive pulmonary disease, and decrease lung injury and certain anti-lung cancer effects (**Table 7**).

**Table 7:** Effects on respiratory diseases.

Disease model	Type	Solvent composition of extract	Animal/cell	Signaling pathway	Major findings	References
Pulmonary fibrosis	*In vivo* + *in vitro*	Asiatic acid	Male C57BL/6, human lung fibroblasts MRC-5 cells and mouse lung epithelial cells MEL-12	–	Collagen levels ↓, inspiratory resistance ↓, expiratory resistance ↓, collagen I ↓, collagen III ↓, α-SMA ↓, Vimentin ↓, E-cadherin ↑, TGF-β1 ↓, fibronectin ↓, p-ERK1/2 ↓, IL-1β↓, IL-18 ↓, IL-6 ↓, TNF-a ↓, NLRP3 ↓	(Dong et al., 2017)
Chronic obstructive pulmonary disease	*In vivo*	Asiatic acid	Male C57BL/6	NF-κB, MAPKs	Neutrophils ↓, macrophages ↓, NE ↓, TNF-α ↓, IL-6 ↓, MCP-1 ↓, p-ERK ↓, p- p38 ↓, p-JNK ↓, p-p65 ↓, p-IkB ↓, HO-1 ↑, SOD3 ↑	(Lee et al., 2016)
Lung cancer	*In vitro* and *in vivo*	Asiatic acid	Human A549 and H1299 lung cancer cell lines and mouse Lewis lung cancer (LLC) cells, C57BL/6J mice	–	Caspase-9 ↑, caspase-3 ↑, LC3-I ↓, LC3-II ↑, p62 ↓, tumor volume ↓	(Wu et al., 2017)
Acute lung injury	*In vitro* and *in vivo*	Asiaticoside	Male BALB/c mice, RAW 264.7 mouse macrophage cell line	NF-κB	TNF-α ↓, IL-6 ↓, p-IκB ↓, p-p65 ↓	(Qiu et al., 2015)
Lung cancer	*In vitro*	*C. asiatica* semi-purified fraction-3 (C3)	Cancerous lung A549 cells	–	MDA ↑, IROS ↑, GSH ↑, GSSG ↓, Nrf-2 ↑, GPx ↑, SOD ↑, LDH ↑, caspase-8 ↑, caspase-9 ↑, caspase-3/7 ↑, ATP ↓, p53 ↓, Bax↑, Bcl-2 ↓, HSP-70 ↑	(Naidoo et al., 2017b)
Lung cancer	*In vitro*	Asiatic acid	Human A549 lung cancer cell line	–	Migration ↓, invasion ↓, Snail ↓, E−cadherin ↓, N−cadherin ↓, vimentin ↓	(Cui et al., 2019)

NLRP3, PYD domains-containing protein 3; NE, neutrophil elastase; LC3, light chain 3; IROS, intracellular reactive oxygen species; LDH, lactate dehydrogenase; HSP-70, heat shock protein -70.

Pulmonary fibrosis can be induced by a variety of injuries to the lung. It is characterized by fibroblast/myofibroblast activation and excessive extracellular matrix accumulation, leading to a progressive organ dysfunction mainly including varying degrees of inflammation and fibrosis (Selman and Pardo, 2002; Thannickal et al., 2004). By reducing collagen accumulation, pulmonary fibrosis can be effectively improved through TGF-β1 and NLRP3 pathways and decreasing the levels of inflammation factors (Nie et al., 2017; Tian et al., 2017). Chronic obstructive pulmonary disease (COPD) is a frequently progressive inflammatory disease of the respiratory tract, alveoli, and microvasculature. The pathological mechanism of COPD is that airway epithelial cell damage triggers nonspecific inflammatory responses by releasing endogenous intracellular molecules or molecular patterns associated with danger. Impaired immune regulation may play a major role in COPD (Rabe and Watz, 2017). For the treatment of COPD, it is generally recommended to use appropriate long-acting maintenance bronchodilators and inhaled corticosteroids; pulmonary rehabilitation can also relieve symptoms (Riley and Sciurba, 2019). However, corticosteroid treatment has certain side effects. Lung cancer is the most common cause of cancer-related mortality in the world. Different treatment methods for lung cancer are generally selected according to the stages, such as surgery, radiation therapy, molecular targeted therapy, and immunotherapy (Hirsch et al., 2017; Quaratino et al., 2017). Acute lung injury (ALI) is a systemic inflammation of the lungs manifested as hypoxia, edema, and pulmonary infiltrates present in the chest cavity. ALI is characterized by (1) epithelial and vascular permeability increased, (2) hypercoagulation and insufficient fibrinolysis, and (3) inflammation and immune regulation (McVey et al., 2012).

This review found that pretreatment with asiatic acid can inhibit bleomycin-induced lung injury and fibrosis in mice. It can down-regulate the expression of pro-inflammatory factors, inhibit inflammatory cells infiltration and expression of transforming growth factor-β1. In a mouse model, lung inflammation was induced by exposure to cigarette smoke, oral administration of asiatic acid reduced the excessive production of mucus in lung tissues, inhibited the release of pro-inflammatory factors, and induce the expression of HO-1, which may become a potential drug for the treatment of COPD by regulating key progressions (Lee et al., 2016; Dong et al., 2017). The common possible mechanism was that asiatic acid could reduce the level of related inflammation factors (IL-6 ↓, TNF-a ↓). In addition, asiatic acid can inhibit collagen deposition in lung fibrosis diseases (Lee et al., 2016; Dong et al., 2017). Cell and animal experiments found that asiatic acid could reduce tumor volume, tumor migration, and differentiation. Furthermore, it also has the ability to promote tumor cell apoptosis (caspase-9 ↑, caspase-3 ↑) (Wu et al., 2017; Cui et al., 2019). Therefore, asiatic acid may be a potential therapeutic drug for lung cancer. As for asiaticoside, the review found that it can reduce the inflammatory infiltration caused by lipopolysaccharide in a dose-dependent manner, and inhibit the inflammatory response in lung tissue by inhibiting the NF-κB signaling pathway(TNF-α ↓, IL-6 ↓, p-IκB ↓, p-p65 ↓), which can be an effective preventive agent for ALI (Qiu et al., 2015).

Therefore, the present study showed that the effective components of *C. asiatica* on respiratory diseases were asiatic acid and asiaticoside, and the major mechanism was anti-inflammation; this was also consistent with the pathological mechanism of the aforementioned diseases. Moreover, it is worth paying close attention to the potential therapeutic effect of *C. asiatica* and asiatic acid on lung cancer. Also, the mechanisms of promoting apoptosis and inhibiting differentiation of tumor cells are worthy of further exploration.

### Effects on Gynecological Diseases

*C. asiatica* can effectively improve endometriosis and relief pelvic inflammatory disease, as well as exert anti-ovarian cancer and anti-breast cancer functions (**Table 8**).

**Table 8:** Effects on gynecological diseases.

Disease model	Type	Solvent composition of extract	Animal/cell	Signaling pathway	Major findings	References
Pelvic inflammation	*In vivo*	Asiatic acid	Female SD rats	NF−κB	IL−1β↓, IL−6↓, TNF−α↓, NLRP3↓, SOD ↑	(Kong et al., 2019)
Ovarian cancer	*In vitro*	Asiatic acid	Ovarian cancer cell lines (SKOV3 and OVCAR-3)	PI3K/Akt/mTOR	Caspase-9 ↑, caspase-3 ↑, PARP ↑, Bax↑, Bcl-2 ↓, p-PI3K ↓, p-Akt ↓, p-mTOR ↓, CDK2 ↓, CDK4 ↓, CDK6 ↓, cyclin D1↓, cyclin E ↓	(Ren et al., 2016)
Breast cancer	*In vitro*	*Centella asiatica*	MCF-7 breast cancer cell line	–	Caspase-3 ↑, caspase-9 ↑	(Fard et al., 2018)
–	*In vitro*	Asiatic acid	Parthenogenetic embryos		ROS ↓, GSH ↑, MMP ↑, blastocyst formation ↑, SOD-1 ↑, COX-2 ↑, caspase-9 ↓	(Qi et al., 2020)
Endometriosis	*In vitro*	Asiatic acid	Endometrial epithelial cells	TLR4/NF-κB	TNF-α ↓, IL-1β ↓, NO ↓, PGE2 ↓, TLR4 ↓, p-IκBα ↓, p- p65 ↓, PPARγ ↑	(Cao et al., 2018)

PARP, antipoly (ADP-ribose) polymerase; CDK, cyclin-dependent kinase; PGE2, Prostaglandin E2; PPARγ, peroxisome proliferator-activated receptor γ.

Pelvic inflammatory disease is a microbial infection of the upper reproductive tract. Its major complications are infertility, chronic pelvic pain, rupture of a renal tubular ovarian abscess, and ectopic pregnancy. Western medicine uses antibiotics to effectively control the symptoms (Jaiyeoba et al., 2011; Bugg and Taira, 2016). However, no conclusive evidence indicated that antibiotic treatment for the pelvic inflammatory disease was safer or more effective than other methods (Savaris et al., 2017). Endometriosis is a common inflammatory disease often accompanied by pelvic pain and infertility. Generally, surgeries are conducted to treat endometriosis, but accumulating evidence suggest the use of plant-based drugs for the treatment, and these medicines usually alleviate the symptoms *via* their anti-inflammatory, anti-oxidant, anti-proliferation, and anti-apoptotic effects (Audebert, 2018; Falcone and Flyckt-Rebecca, 2018; Bina et al., 2019). Ovarian cancer and breast cancer are the top two cancers in women (Anastasiadi et al., 2017; Stewart et al., 2019). Surgery, radiotherapy, chemotherapy, and other treatments are often associated with various complications. Therefore, exploring new therapeutic agents is particularly important.

**Table 8** shows that asiatic acid can efficaciously treat pelvic inflammation. It has potential therapeutic effects on endometriosis and ovarian cancer (Ren et al., 2016; Cao et al., 2018; Kong et al., 2019). The main mechanism is reduction in the production of inflammatory body NLRP3 and inflammatory factors (IL−1 β, IL−6, TNF−α), inhibition of the NF−κB signaling pathway, which regulates the production of inflammatory factors, and thus alleviation of pelvic inflammation. Cell experiments confirmed that asiatic acid can effectively improve the symptoms of endometriosis. The main mechanism is inhibition of the NF-κB pathway to reduce the production of inflammatory factors (TNF-α ↓, IL-1β ↓, p-IκBα ↓, p- p65 ↓) (Cao et al., 2018). The potential therapeutic value of asiatic acid for ovarian cancer is mainly reflected in that it can promote the apoptosis of ovarian cancer cells and inhibit the growth of ovarian cancer cells by affecting the cell cycle progression (Ren et al., 2016). In addition, studies found that asiatic acid improved the developmental ability of early embryos in pigs; the main underlying mechanism was amelioration of oxidative stress and downregulation of the expression of apoptosis-related genes (Qi et al., 2020). The potential therapeutic value of *C. asiatica* for breast cancer mainly reflected in the promotion of apoptosis of breast cancer cells (Fard et al., 2018).

Therefore, this study concluded that the therapeutic effect of *C. asiatica* on the gynecological diseases mainly reflected in the improvement in inflammation. The main research component was asiatic acid, which worked by affecting apoptosis, reducing the production of inflammatory factors and influencing the cell cycle progression. Therefore, asiatic acid may be a potential agent in the treatment of gynecological diseases, and further clinical trials are needed to verify its efficacy.

### Effects on Rheumatoid Arthritis

Animal and cell experiments confirmed that madecassoside exerted an anti-rheumatoid effect (**Table 9**). Rheumatoid arthritis (RA) is a chronic inflammatory joint disease that usually affects women and elderly people; the main pathological change is persistent synovitis. If not controlled well, it can lead to joint deformities and other diseases and decrease patients’ quality of life (Scott et al., 2010; Smolen et al., 2016). Studies confirmed that TNF-α is a powerful proinflammatory cytokine overexpressed in the synovium of patients with RA, and reducing TNF-α production can effectively improve the symptoms of rheumatoid arthritis(Marsal and Juliá, 2010). In addition, matrix metalloproteinase (MMP)-13 is a specific protein associated with RA, and may be involved in the physiological remodeling of synovial tissue (Konttinen et al., 1999).

**Table 9:** Effects on rheumatoid arthritis.

Disease model	Type	Solvent composition of extract	Animal/cell	Signaling pathway	Major findings	References
Rheumatoid arthritis	*In vivo*	Madecassoside	Female Wistar rats	–	TNF-α ↓, IL-1b ↓, IL-6 ↓, IFN-γ ↓, IL-17 ↓, IL-10 ↑	(Wang et al., 2015)
Rheumatoid arthritis	*In vivo + in vitro*	Madecassoside	Adjuvant-induced arthritis model rats, fibroblast-like synoviocytes (FLS) cells	NF-κB/MMP-13	MMP-13 mRNA↓, NF-κB ↓	(Yu et al., 2018)

The pharmacological study on madecassoside demonstrated that it can effectively lessen the related inflammatory factors in arthritis model rats (TNF-α ↓, IL-1b ↓, IL-6 ↓, IFN-γ ↓, IL-17 ↓). Animal experiments have confirmed that oral madecassoside (30 mg/kg) can significantly reduce the symptoms of arthritis, and can inhibit the secretion of inflammatory cytokines. However, *in vitro* experiments have found that madecassoside and madecassic acid, the main metabolite of madecassoside, cannot influence the secretion of inflammatory cytokines. It was subsequently suggested that madecassoside may exhibits anti-arthritis potency through affecting the secretion of IL-10 from Foxp3^+^ cells in lamina propria of intestine, thus regulates the immune function of rats with collagen-induced arthritis (Wang T. et al., 2015). The results of madecassoside pharmacokinetics experiments are poor, but it has significant bioavailability, can effectively reverse adjuvant-induced arthritis, and inhibit the migration and invasion of fibroblast-like synovial cells, however, it has no effect on cell proliferation. Wei-GuangYU et al. pointed out that madecassoside may have anti-arthritis activity by inhibiting the NF-κB/MMP-13 pathway (Yu et al., 2018).

In summary, madecassoside as a triterpene component of *C. asiatica*, may have anti-arthritis effect, and the underlying mechanism is reduction in the level of inflammatory factors.

### Effects on Other Diseases

Besides the aforementioned diseases, the preclinical studies on *C. asiatica* and its triterpenoids reported its other positive effects listed in **Table 10**, including (1) protecting retinal blood vessels, (2) reducing toxic and side effects of drugs, (3) reducing drug resistance, and (4) promoting periodontal tissue regeneration. *C. asiatica* could also alleviate oral submucous fibrosis, sepsis, migraine, glaucoma, periodontitis, leukemia, and osteolytic bone diseases.

**Table 10:** Effects on other diseases.

Disease model	Type	Solvent composition of extract	Animal/cell	signaling pathway	Major findings	References
Leukemia	*In vitro*	*C. asiatica* ethanolic leaf extract	THP-1 cells	–	Cell viability ↓, TNF-α ↑, IL-1β ↓, IL-6 ↓, IL-10 ↑, GSH ↑, caspase-9, caspase-3/7) ↓, ATP ↓	(Naidoo et al., 2017a)
Ischemic retinopathies	*In vitro*	Madecassic acid	hRMECs	PERK/eIF2a	Cell viability ↑, SOD ↑, GSH-PX ↑, LDH ↓, MDA ↓, caspase-3 ↓, caspase-9 ↓, Bax/Bcl-2 ↓, GRP78 ↓, CHOP ↓, ATF6 ↓, IRE1a ↓, PERK ↓, eIF2a ↓	(Yang et al., 2016)
Doxorubicin (DXR)-induced organ toxicities	*In vivo*	Asiatic acid	Wistar rats	–	CK-MB ↓, LDH ↓, SGPT ↓, SGOT ↓, BUN ↓, creatinine ↓, SOD ↑, GSH ↑, LPO ↓, Nrf2 ↑	(Kamble and Patil, 2018)
Drug resistance	*In vitro*	Asiatic acid	Cisplatin-resistant A549/DDP cells	NF-kB, MAPK/ERK	DDP resistance ↓, DDP sensitivity ↑, MDR1 ↓, p65 ↓, Akt ↓, YB1 ↓, p-ERK1/2 ↓, p-JNK ↓, p-p38 ↓, p-IkBα↓	(Cheng et al., 2018)
Oral submucous fibrosis	*In vitro*	Ethanolic extract of CA, Asiatic acid	Primary human buccal fibroblasts(HBF)	TGFβ/Smad	TGFβ1 ↓, COL1A2 ↓, COL3A1 ↓, extracellular matrix (ECM) deposition ↓	(Adtani et al., 2017)
–	*In vitro*	ECa 233	RAW264.7 macrophages	MAPK	ROS ↓, NO ↓, PGE2 ↓, TNF-α ↓, IL-1β ↓, COX-2 ↓, iNOS ↓	(Sukketsiri et al., 2019)
–	*In vitro*	Asiatic acid	HepG2 cells	Nrf2	ROS↓, apoptosis↓, Nrf2↑, Keap1↓, HO-1↑, NQO-1↑, GCLC↑	(Qi et al., 2017)
Sepsis	*In vivo + In vitro*	Asiatic acid	BALB/c mice, RAW264.7 cells	Notch	neutrophil infiltration↓, ALT ↓, BUN ↓, IL-1β ↓, IL-6 ↓, NO ↓, Notch3 ↓, Dll4 ↓, ROS ↓, MMP ↓, ATP ↓	(Yuyun et al., 2018)
Migraine	*In vivo*	Asiaticoside (AS)-based standardized extract of *Centella asiatica*	Wistar rats	–	Hyperalgesia ↓, 5-HT ↑, vocalization ↓, pain latency ↑	(Bobade et al., 2015)
Glaucoma	*In vivo*	Asiatic acid	Wistar rats,	–	RGCs ↑, Bcl-2 ↑, Bax ↓, caspase-3 ↓	(Huang et al., 2018)
–	*In vitro + in vivo*	Methanol extract of CA	Swiss albino male mice	MAPK	Lipid peroxide ↓, superoxide ↓, hydroxyl radicals ↓, RNS ↓, IL-10 ↑, IL-1 ↓, MCP-1 ↓, INF-γ ↓, TNF-β ↓, MAPK 14 ↓	(Viswanathan et al., 2019)
Periodontitis	*In vivo + in vitro*	Asiatic acid	Male SD rats, HGFs, RAW264.7 cells	NF-κB	PGE2 ↓, NO ↓, IL-8 ↓, IL-6 ↓, p-p65 ↓, p-IκBα ↓, PPAR-γ ↑	(Hao et al., 2017)
–	*In vitro*	Asiaticoside	hPDL cells	Wnt/β-catenin	OSX ↑, WNT3A ↑, AXIN2 ↓, DMP 1↑	(Fitri et al., 2018)
Toxic side effects	*In vivo*	Ethanolic extraction of CA	Male Wistar albino rats	–	WBC ↓, TBARS ↓, SOD ↑, CAT ↑, GSH ↑, TSP ↑, albumin ↑, ALT ↓, AST ↓, ALP ↓, globulin ↓, total bilirubin ↓, urea ↓, creatinine ↓	(Ghosh et al., 2017)
Osteolytic bone diseases	*In vitro*	Asiaticoside	Bone marrow macrophages (BMMs)	NF‐κB	TRAcP-positive cells ↓, Ctsk ↓, Nfatc1 ↓,V‐ATPase d2 ↓, NFAT ↓, cFos↓, Ca^2+^ oscillation ↓, IκB‐α ↑, p-ERK ↓	(He et al., 2019)

GRP78, glucose-regulated protein 78; CHOP, C/EBP homologous transcription factor; ATF6, activating transcription factor 6; IRE1a, inositolrequiring kinase/endonuclease 1 alpha; PERK, phosphorylation of pancreatic ER stress kinase; eIF2a, eukaryotic initiation factor 2 alpha; CK-MB, Creatine kinase-MB; BUN, blood urea nitrogen; SGPT, serum glutamic pyruvic transaminase; SGOT, serum glutamic-oxaloacetic transaminase. DDP, cisplatin; MDR, multidrug resistance; YB1, Y-box binding protein 1; COL1A2, collagen 1A2; COL3A1, collagen 3A1; NQO-1, NAD(P)H: quinone oxidase; GCLC, glutamyl cysteine ligase catalytic subunit; Dll4, delta-like ligand; 5-HT, 5-hydroxytryptophan; RGCs, retinal ganglion cells; RNS, reactive nitrogen species; OSX, osterix; ALP, AXIN2, axis inhibition protein 2;DMP 1, dentin matrix protein1; TSP,total serum protein; ALP,alkaline phosphatase; TRAcP, tartrate resistant acid phosphatase; Ctsk, cathepsin K; Nfatc1, V‐ATPase d2;, vacuolar‐type H^+^‐ATPase V0 subunit d2; NFAT, nuclear factor of activated T-cells.

Moreover, the *C. asiatica* extract has a positive significance for leukemia, oral submucous fibrosis, migraine, and toxic side effects. Leukemia is mainly affected by increased activity of oxidative scavengers. *In vitro* experiments found that the activity of leukemic THP-1 cells treated with *C. asiatica* ethanolic leaf extract (CLE) decreased by 28.404%. Also, the levels of IL-1β and IL-6 decreased, but the level of IL-10 increased, which may reduce the cytokine-induced tumor immunosuppressive activity, cancer progression and cancer cachexia syndrome. Moreover, *C. asiatica* can also activate exogenous apoptosis pathways in THP-1 cells, and may reduce the proliferation of THP-1 cells by cutting down the levels of ATP. Therefore, it may be effective in treating leukemia cachexia (Naidoo et al., 2017a). In addition, *C. asiatica* was found to be effective in reversing the hyperalgesia and 5-hydroxytryptophan levels in the brain of migraine animal models. Compared with the positive control (sumatriptan, 42 mg kg^−1^), the oral treatment agent [asiaticoside (AS)-based standardized *C. asiatica* extract (30 mg kg^−1^, 7 days)] was effective in reducing nociception in rats (Bobade et al., 2015). Furthermore, an *in vitro* experiment showed that *C. asiatica* also downregulated fibrotic markers (TGFb1 ↓, COL1A2 ↓, COL3A1 ↓) to reverse oral mucosal fibrosis caused by arecoline. Therefore, *C. asiatica* might have an anti-fibrotic effect (Adtani et al., 2017). Finally, an *in vivo* experiment found that *C. asiatica* at 100 mg/kg bw effectively reduced the side effects of isoniazid in the treatment of pulmonary tuberculosis. This oral dose restored abnormal indicators to normal or even close to normal levels as reflected by liver and kidney functions (Ghosh et al., 2017).

Asiatic acid can improve the side effects caused by antibiotics, reverse multidrug resistance (MDR), and reduce sepsis. It also has therapeutic potential for periodontitis and glaucoma. An *in vivo* experiment showed that asiatic acid had a visible effect on doxorubicin (DXR)-induced organ toxicities, showing the best effect at 20 mg/kg by affecting the expression of Nrf2 (Kamble and Patil, 2018). Doxorubicin causes the peroxidation of organs and reduces the activity of innate antioxidant factors (Sonawane et al., 2018) while upregulating the expression of Nrf2 effectively promotes antioxidant activity and protects cells from the damage caused by oxidative stress (Qi et al., 2017; Delgado-Wicke et al., 2020). *In vivo* and *in vitro* experiments also found that asiatic acid reduced the levels of inflammatory factors (IL-1β ↓, IL-6 ↓) by affecting the Notch signaling pathway, weakened liver and kidney damage, and improved the survival rate in the experimental sepsis mice model (Yuyun et al., 2018). Asiatic acid may also provide therapeutic latent energy for periodontitis through influencing the NF-κB signaling pathway and reducing the levels of related inflammatory factors (IL-8 ↓, IL-6 ↓, p-p65 ↓, p-IκBα ↓) (Hao et al., 2017). Finally, asiatic acid also has a potential therapeutic effect on glaucoma; it can improve the survival rate of retinal ganglion cells (RGCs) in the glaucoma rat model. More importantly, it can increase the level of anti-apoptotic factors (Bcl-2 ↑) while reducing the level of apoptotic factors (Bax ↓, caspase-3 ↓) (Huang et al., 2018).

Madecassic acid can ameliorate ischemic retinopathy. A cell experiment showed that madecassic acid reduced apoptosis and endoplasmic reticulum stress for hypoxia-induced human retinal microvascular endothelial cells (hRMECs). It also reversed cell dysfunction through affecting the oxidative stress of cells under hypoxic conditions (Yang et al., 2016).

Interestingly, *in vitro* experiments found that asiaticoside effectively promoted the osteogenic differentiation of human periodontal ligament and inhibited receptor activator of nuclear factor kappa B ligand (RANKL)-induced formation of osteoclasts, indicating its therapeutic potential for periodontal tissue regeneration and osteolytic diseases (Fitri et al., 2018; He et al., 2019).

In summary, *C. asiatica* and its triterpenoids have broad therapeutic potential. The specific mechanism mainly involves the following four aspects: (1) anti-inflammatory; (2) antioxidant; (3) anti-apoptosis; and (4) anti-fibrosis.

### Clinical Trials of *C. asiatica*

Clinical studies (**Table 11**) found that *C. asiatica* effectively improved the cognitive function of stroke patients. Patients were divided into 3 groups and administered with 1,000 mg/day, 750 mg/day *C. asiatica* extract, and 3 mg/day folic acid, respectively. The patients were treated at the acute phase of stroke infarction for 6 weeks. The cognitive function of the patients was evaluated by the MoCA-Ina test. The 1,000 mg/day treatment group scored highest among the three. No significant difference was noticed in AST and ALT levels when compared with baselines. As shown in **Table 11**, patients had different degrees of side effects, such as constipation, skin itching and abdominal distension.). Regarding the safety and pharmacokinetics of *C. asiatica* in healthy volunteers from Thailand, *Phanit Songvut et al*. pointed out that daily oral doses (single or multiple) of 250 mg and 500 mg are safe for patients, and reported just mild to moderate adverse events. The pharmacokinetics of the human studies are inconsistent with animal studies. The main manifestation is that there is β-glycosidase in the human intestine, which can hydrolyze glycosides, while the animal’s β-glycosidase cannot hydrolyze these glycosides. Because asiaticoside and madecassoside are glycosides, they cannot be easily absorbed by the gastrointestinal tract. Therefore, they are hydrolyzed into asiatic acid and madecassic acid by β-glycosidase, then these aglycones exert biological functions. The plasma concentration-time curve reflects that asiaticoside and madecassoside reach Tmax within 1–2 h after oral administration, while their metabolites asiatic acid and madecassic acid reach Tmax within 3 h, and accompanied by downward trend of parent compound (Songvut et al., 2019). Madecassoside was considered to be a substrate of efflux transporters, which may influence the drug absorption in the gastrointestinal tract. In another study, linked-rat models showed that enterohepatic circulation participates in the absorption and utilization of madecassoside (Leng et al., 2013; Anukunwithaya et al., 2017). A randomized, controlled, double-blind clinical trial found that *C. asiatica* have the potential to reduce diabetic neuropathy. Patients were administered capsules containing *C. asiatica* selected triterpenes for 52 weeks, a significant reduction was observed in their total symptom score (TSS). However, almost 67% of the patients in the treatment group experienced at least one adverse event, including transient liver, kidney or gastrointestinal dysfunction, but these symptoms resolved on their own (Lou et al., 2018). Another randomized controlled double-blind trial found that *C. asiatica* cream containing 5.12% asiaticoside and 5.1% madecassoside can be completely absorbed by the skin and effectively improve pigmentation and may be used in treating hypertrophic scars (Jenwitheesuk et al., 2018). In a 4-week study of a group of 25 volunteers, a cosmetic formula containing the *C. asiatica* extract was applied on the forearm twice a day, which were prepared into emulsion and hydrogel preparations containing 2.5 and 5% *C. asiatica* extract respectively. Revealing that this formula increased the hydration status of skin surface, reduced epidermal water loss, and exerted an anti-inflammatory effect. In addition, the hydration and epidermal barrier function of the subjects in emulsion formulation group was better than that of hydrogel formulation group. Therefore, *C. asiatica* can be used in moisturizing cosmetic formulations (Ratz-Lyko et al., 2016). Clinical studies also confirmed the effect of *C. asiatica* against generalized anxiety disorder, but the level of evidence was low and the number of patients was rather small (Jana et al., 2010). In addition, a 21-day prospective randomized control study found that *C. asiatica* extract can effectively promote wound healing in diabetic patients without any serious side effects (Paocharoen, 2010). Above all, clinical trials have found that *C. asiatica* can improve cognitive function, relieve anxiety, promote wound healing, and has effect for skin care, but these effects require more clinical studies with higher levels of evidence need to be performed urgently to validate the findings.

**Table 11:** Clinical trials of *C. asiatica*.

Diseases	Extract/Formulation	Design	Dose regimen	Duration	Case/Control	Main outcome	Side effects	Follow-up	References
Vascular cognitive Impairment	Gotu kola extract	–	750 mg/day or 1000 mg/day	6 weeks	17/17/14	MoCA-Ina score↑	Constipation, itching, bloated feeling	6 weeks	(Farhana et al., 2016)
Diabetic neuropathy	*Centella asiatica* selected triterpenes	RCT	120 mg, 180 mg, 240 mg	52 weeks	21/22	TSS ↓, paresthesia↓, burning sensation↓	Transient abnormal liver and kidney function or gastrointestinal symptoms	Not mentioned	(Lou et al., 2018)
Scar	*Centella* cream	RCT	Unknown	12 weeks	30/30	Vancouver scores↑, pigmentation ↓	Allergic dermatitis	Not mentioned	(Jenwitheesuk et al., 2018)
–	Cosmetic formulations of *Centella asiatica* extract	–	Unknown	4 weeks	25/0	Skin moisture↑, TEWL↓, skin redness↓	Not mentioned	Not mentioned	(Ratz-Lyko et al., 2016)

RCT, Randomized controlled trial; TSS, total symptom score; TEWL, transepidermal water loss.

### Safety and Toxicity of *C. asiatica*

As for the safety of *C. asiatica* extract, clinical trials have shown that 250 mg and 500 mg of standard extract were well tolerated in single and multiple oral doses. Modern pharmacological tests showed that the interaction potential of *C. asiatica* biologically active compounds with CYP isoenzymes is negligible, and the heavy metal content in the extract is within the allowable range (Kar et al., 2017). Animal experiments have found that *C. asiatica* extract has anti-spermogenic and anti-fertility effects on the reproductive system of male rats (Yunianto et al., 2010). Hematological parameters and histopathology in acute oral toxicity study, sub-chronic toxicity study and mutagenicity study have confirmed that *C. asiatica* extract is safe in rats. Also, *C. asiatica* extract did not show any dose-related adverse effects in Ames test (Deshpande et al., 2015). However, there are case reports that three women developed jaundice after taking *C. asiatica* for 30, 20, and 60 days, they were clinically diagnosed with granulomatous hepatitis, and their symptoms improved after the drug was stopped (Jorge and Jorge, 2005). About the triterpenoids of *C. asiatica*, the previous clinical trials have showed that emulsions and capsules, which contain several major triterpenoids have different degrees of side effects for patients, but they all relieve without any medical interventions. Although pre-clinical studies of *C. asiatica* have found that it has a wide range of pharmacological effects and demonstrated the safety of it, but considering the bad reports in few clinical cases, rigorous research is recommended for the exploration of clinical dosages with highest safety.

## Conclusions and Perspectives

*C. asiatica* is an herb used in traditional Chinese medicine. Its main effective components are asiaticoside, asiatic acid, madecassoside, and madecassic acid. As mentioned earlier, *C. asiatica* and its triterpenoids have a wide range of medicinal values. *In vivo* and *in vitro* studies showed that *C. asiatica* and its triterpenoids had therapeutic and relieving effects on multi-system diseases. The *C. asiatica* extract effectively relieves sleep deprivation, AD, type 2 diabetes mellitus (T2DM), hyperlipidemia, gestational diabetes, baldness, atopic dermatitis, wound, drug-induced liver toxicity, liver injury, gastric mucosal injury, gastric ulcers, breast cancer, leukemia, oral submucous fibrosis, migraine, and so forth. Asiatic acid effectively relieves cognitive impairment, Alzheimer’s disease, Parkinson ‘s disease, obesity, renovascular hypertension, transverse aortic constriction, myocardial ischemia/reperfusion (MI/R) injury, atherosclerosis, liver fibrosis, acute pancreatitis, colon carcinogenesis, hepatocellular carcinoma, pulmonary fibrosis, lung cancer, pelvic inflammatory, ovarian cancer, endometriosis, sepsis, periodontitis, and so forth. The preclinical studies on asiaticoside found that it has therapeutic potential for the following diseases: hemiparkinsonism, Alzheimer’s disease, cerebral ischemia, skin wound, pulmonary hypertension, atherogenesis, ALI, osteolytic bone diseases, and so forth. Pharmacological studies found that madecassoside had potential therapeutic effect against osteoporosis, acne, vitiligo, RA, and so forth. Madecassic acid had a positive therapeutic effect on ischemic retinopathies. The preclinical study on *Centella asiatica* mainly focused on the extract of *C. asiatica* and asiatic acid. For diseases, neurological and skin diseases are mostly investigated. However, the impact on other diseases also needs further in-depth exploration.

**Figure 3:**
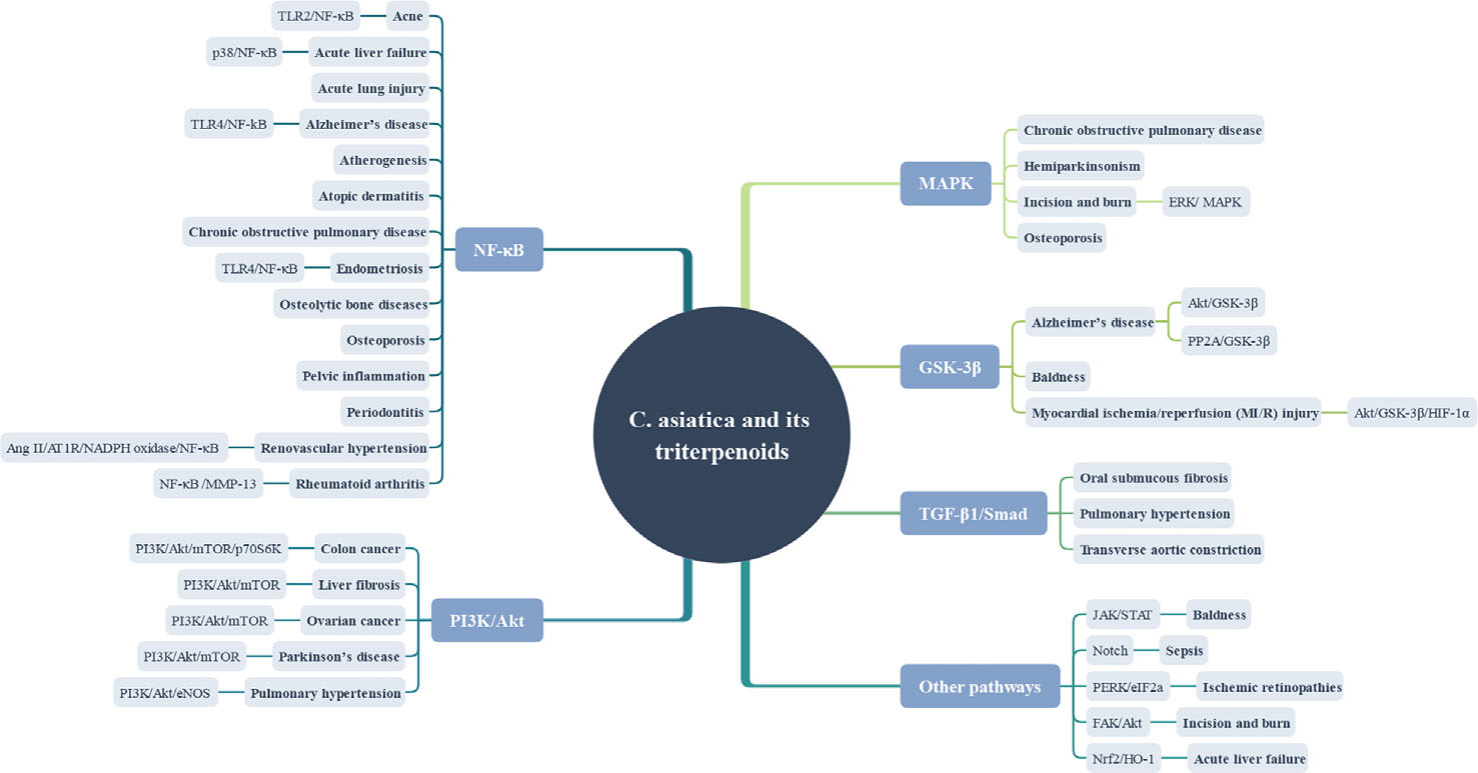
Therapeutic potential and mechanisms of action of *C. asiatica* and its triterpenes.

The occurrence of inflammatory response, oxidative stress, apoptosis, and mitochondrial dysfunction is closely related to various diseases. *C. asiatica* and its triterpenoids can be used in many medical situations because they have anti-inflammatory and anti-apoptotic effects, relieve oxidant stress, and improve mitochondrial function (**Figure 3**). Thus further, *C. asiatica* may also be applied to diseases not mentioned in this study *via* the same pathological mechanism, and this hypothesis needs in-depth investigation for verification.

Last but not least, this study summarized the *in vitro* and *in vivo* studies on *C. asiatica* and its triterpenoids and listed the significant changes in factors for each disease, providing a reference for subsequent studies. However, only four clinical studies were included, the level of clinical evidence was weak, and more clinical trial supplements were needed. More importantly, the pharmacokinetics of this TCM needs further improvement. Also, the toxic and side effects of the drug, the effective therapeutic dose, and the standardization of the agent still need to be explored. The pharmacological effects of *C. asiatica* should be explored in detail to provide more rigorous data support for future clinical applications.

## Author Contributions

BS and LW designed the study. BS, LW, YW, MH, and MK reviewed the relevant literature and wrote the manuscript. CZ, YW, LQ, and LW contributed to the scientific writing of the manuscript. BS, MG, and TL revised the manuscript. All authors contributed to the article and approved the submitted version. BS, LW, YW, CZ, LQ, MH, MK, MG, and TL contributed equally to this study. BS, LW and YW contributed equally to this study and share first authorship.

## Funding

This paper was supported by the International Cooperation Base for the Prevention and Treatment of Chronic Diseases by Traditional Chinese Medicine. No. GZYYGJ2019034.

## Conflict of Interest

The authors declare that the research was conducted in the absence of any commercial or financial relationships that could be construed as a potential conflict of interest.
