# Supplementary figures and images for: Therapeutic Potential of Centella asiatica and Its Triterpenes: A Review
Source: Front Pharmacol. 2020 Sep 4;11:568032. doi: 10.3389/fphar.2020.568032 (PMC7498642; doi:10.3389/fphar.2020.568032)

Supplementary Material


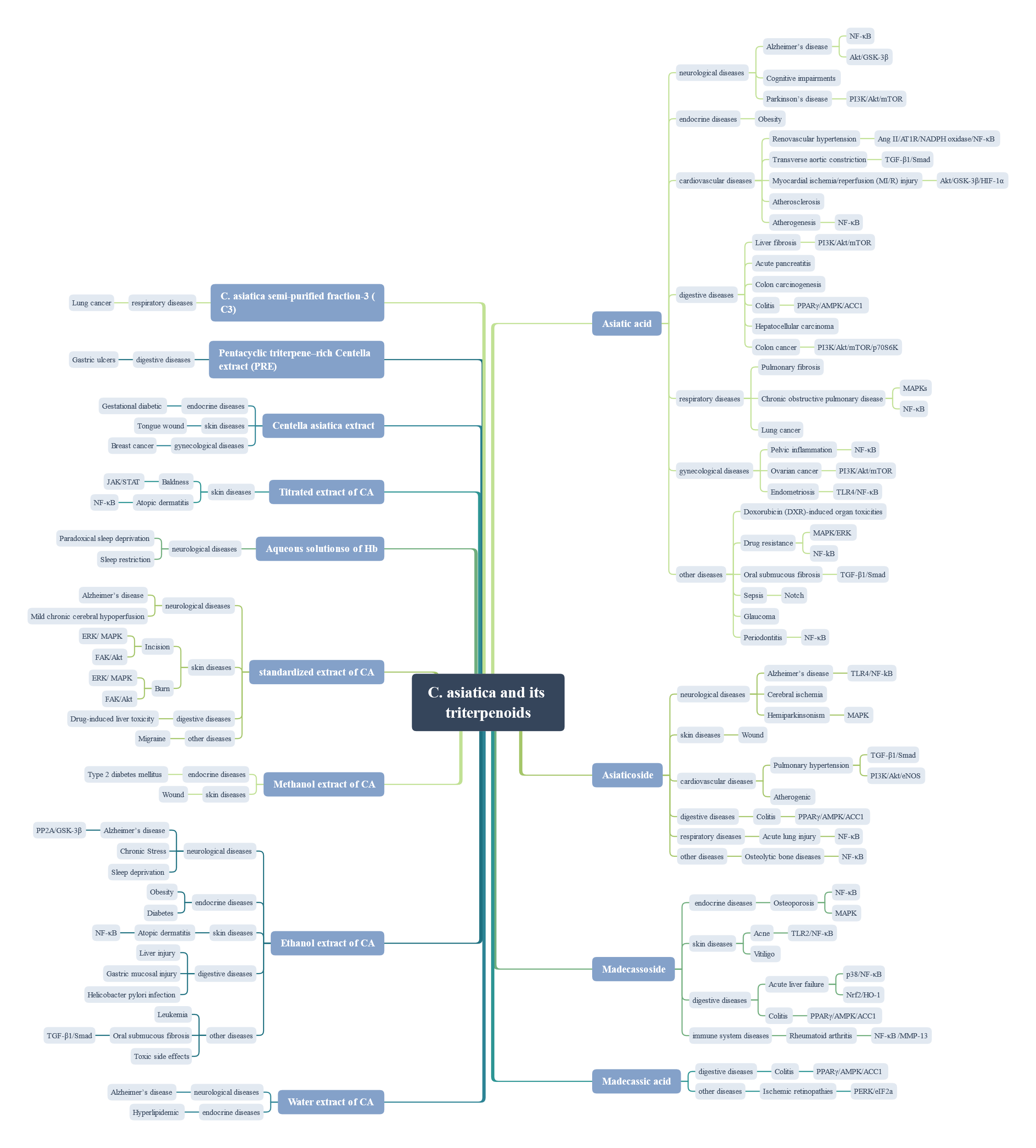

Supplement: Supplementary file 1 [file DataSheet_1.docx]
